# A Re-examination of the Selection of the Sensory Organ Precursor of the Bristle Sensilla of *Drosophila melanogaster*

**DOI:** 10.1371/journal.pgen.1004911

**Published:** 2015-01-08

**Authors:** Tobias Troost, Markus Schneider, Thomas Klein

**Affiliations:** Institut fuer Genetik, Heinrich-Heine-Universitaet Duesseldorf, Duesseldorf, Germany; New York University, United States of America

## Abstract

The bristle sensillum of the imago of *Drosophila* is made of four cells that arise from a sensory organ precursor cell (SOP). This SOP is selected within proneural clusters (PNC) through a mechanism that involves Notch signalling. PNCs are defined through the expression domains of the proneural genes, whose activities enables cells to become SOPs. They encode tissue specific bHLH proteins that form functional heterodimers with the bHLH protein Daughterless (Da). In the prevailing lateral inhibition model for SOP selection, a transcriptional feedback loop that involves the Notch pathway amplifies small differences of proneural activity between cells of the PNC. As a result only one or two cells accumulate sufficient proneural activity to adopt the SOP fate. Most of the experiments that sustained the prevailing lateral inhibition model were performed a decade ago. We here re-examined the selection process using recently available reagents. Our data suggest a different picture of SOP selection. They indicate that a band-like region of proneural activity exists. In this proneural band the activity of the Notch pathway is required in combination with Emc to define the PNCs. We found a sub-group in the PNCs from which a pre-selected SOP arises. Our data indicate that most imaginal disc cells are able to adopt a proneural state from which they can progress to become SOPs. They further show that bristle formation can occur in the absence of the proneural genes if the function of *emc* is abolished. These results suggest that the tissue specific proneural proteins of *Drosophila* have a similar function as in the vertebrates, which is to determine the time of emergence and position of the SOP and to stabilise the proneural state.

## Author Summary

The sensory organ precursor cell (SOP) that forms the mechanosensory bristles of the adult PNS of *Drosophila* is a paradigm to study neural precursor determination. The current model states that the SOP is selected in proneural clusters (PNCs) defined through the expression of the proneural genes. The selection occurs through lateral inhibition mediated by the Notch signalling pathway. The SOP is pre-selected by differential expression of Extramacrochaetae (Emc), the only member of the Id proteins in *Drosophila*, which inactivates the proneural factors. We have re-examined the selection process using novel markers and mutants. Our data suggest a different picture of SOP selection. We discovered a band–like region of varying proneural activity where the peaks constitute the proneural clusters. Within the PNC, a subgroup exists from which the SOP arises. The Notch pathway has two distinct functions in the subgroup and in the rest of the band. We show that so far one unappreciated essential role of the proneural genes is the neutralisation of the activity of Emc. Our data suggest that the selection of the SOP is more similar to neural selection in vertebrates than previously anticipated.

## Introduction

The body of the imago of *Drosophila melanogaster* is covered with mechanosensory bristles, called macrochaetae (MCs) and microchaetae (mcs). In the notum, mcs cover the central regions, whereas the larger MCs arise at precise positions in peripheral regions and form a stereotypic pattern. Both sensilla consist of only four cells, which are the progenies of a single neural precursor cell, termed sensory organ precursor cell (SOP). The SOPs of MCs develop in the wing imaginal disc during the second half of the third larval instar stage in a precise temporal sequence [1]. Its development is a paradigm to study fundamental aspects of the determination of a neural precursor cell (reviewed in [2]).

The SOP is selected within proneural clusters (PNC), which are defined through the expression of tissue-specific proneural genes. In the notum these are *achaete* (*ac*) and *scute* (*sc*), two members of the *achaete*-*scute* complex. Their activity conveys cells into a proneural state from which they can proceed to become SOPs if they reach a threshold level of proneural activity. Concomitant loss of their function results in the loss of all bristles of the notum. They encode transcription factors of the class II bHLH family, have identical expression patterns and function redundantly (bHLH factors and their classification are reviewed in [3]). Class II proteins possess a basic DNA binding domain and a HLH domain that mediates dimerization with the ubiquitously expressed Daughterless (Da), the only class I bHLH protein in *Drosophila*. Class V HLH proteins are antagonists of bHLH factors. They lack a basic DNA binding domain and form non-functional heterodimers with class I and II bHLH factors. Thus, they are negative posttranslational regulators of bHLH transcription factors. The only *Drosophila* class V member is Extramacrochaetae (Emc), which forms inactivating heterodimers with Ac, Sc and Da (reviewed in [4]). Weak alleles of *emc* cause formation of additional MCs in homozygousity. Analysis of the *emc* null alleles in the eye imaginal disc revealed a regulatory loop between Da and Emc, where Da activates expression of Emc and itself and Emc in turn inactivates Da [5]. This loop assures that both factors are expressed at correct levels. Loss of *emc* function causes up-regulation of Da expression. The consequences of this up-regulation for bristle development have not been investigated.

Proneural genes play a similar, but not identical role in mammals (reviewed in [6]): In *Drosophila* the activity of the proneural genes appears to confer a proneural state onto cells and promote neural differentiation, while their mammalian counterpart only promote neural differentiation of neural plate cells, which have adopted a proneural state through other mechanisms.

The SOP is selected among the cells of the PNC by a mechanism that is called lateral inhibition mediated by the Notch signalling pathway (reviewed in [7]). Upon loss of Notch activity, all cells in a PNC adopt the SOP fate, indicating that it prevents them progressing from the proneural to the SOP state. Most genes contributing to Notch signalling produce this neurogenic phenotype upon loss of function and are therefore classified as neurogenic genes. In *Drosophila* Notch is activated by two ligands of the DSL family, Delta (Dl) and Serrate (Ser) (reviewed in [8]). Dl is the main ligand for SOP selection, but Ser has a redundant role [9]. Binding of the ligands to Notch initiates the release of its intracellular domain (NICD) into the cytosol. NICD is transported into the nucleus where it associates with the CSL transcription factor Suppressor of Hairless (Su(H)) to activate the target genes. The release of NICD occurs through two proteolytic cleavages of Notch. A ligand-induced first cleavage (S2) by Kuzbanian (Kuz) creates a membrane inserted intermediate (Notch EXtracellular Truncation (NEXT)), which is cleaved by γ-secretase (S3-cleavage) to release NICD. γ-secretase is a complex consisting of Presenilin (Psn), Anterior pharynx defective 1 (Aph-1), Nicastrin (Nic) and Presenilin enhancer 2.

The membrane-associated E3-ubiquitin ligases Neuralized (Neur) and Mindbomb1 (Mib1) are important for the activity of both DSL ligands [10]. They probably mediate the ubiquitylation of the intracellular domains (ICDs) of Dl and Ser on lysines, which in turn initiates their endocytosis. In *Drosophila* the two ligases have similar functions [9], [11]. In imaginal discs of *Drosophila*, expression of Neur is restricted to the SOP, while Mib1 is ubiquitously expressed, indicating that most DSL signalling probably depends on Mib1 [12]. During SOP selection the main target of the Notch pathway are the members of the *Enhancer of split complex* (*E(spl)-C*), which encode class IV bHLH proteins. These proteins antagonise the activity of the proneural factors to suppress SOP development (reviewed in [13]).

According to the lateral inhibition model, activation of the Notch pathway antagonises a cells ambition to adopt the SOP fate, because it initiates the expression of the *E(spl)-C* genes, which suppress the activity of Ac and Sc (reviewed in [7]). While Notch is expressed ubiquitously, the activity of the pathway is linked to that of the proneural proteins through transcriptional regulation of Dl. Consequently, cells with high proneural activity express high levels of Dl and can send a strong inhibitory signal to their immediate neighbours, which prevents them from adopting the SOP fate. The resulting regulatory feedback-loop automatically selects the SOP: All cells of a PNC initially express similar levels of proneural activity and therefore mutually inhibit each other from adopting the SOP fate through Dl/Notch signalling (S1A Fig.). A small difference in activity of the proneural factors results in a small difference in the expression of Dl among cells of a cluster. The regulatory feedback loop between proneural activity and Dl expression amplifies the initially small difference and transforms it in an all or nothing situation: The result is a cell with high proneural activity and high Dl expression that becomes the SOP and neighbours with no proneural activity that switch fate to become epidermoblasts. Emc appears to contribute to the initial bias of proneural activity, through its differential expression among cells of the PNC [14]. Thus, while all cells initially express similar levels of Ac and Sc, they have different proneural activity due to the differential expression of Emc. The SOP arises at positions of the lowest Emc expression and, hence, highest proneural activity. Note, that the lateral inhibition model predicts changes in the expression of Dl and the activity of the Notch pathway during the selection of the SOP.

One problem of the lateral inhibition model is to explain how the nascent SOP inhibits cells in the PNC that are located more than one cell diameter away, since Dl, as a transmembrane protein, reaches only the next cell. Recent reports provided an explanation, which is based on the observation that SOPs directly contact remote cells through filopodia [15], [16]. These contacts are thought to transfer the Dl signal to these remote cells. However, experimental proof that shows that these contacts mediate inhibition is scarce. Moreover, the selection of SOPs in *Dl Ser* double mutant PNCs where Dl is expressed in all cells at uniform levels occurs normally [9]. A similar observation has been made during the selection of neuroblasts in the embryo [17]. These data suggest that differential expression of Dl, which is a hallmark of the lateral inhibition model, is dispensable for the selection process. A recent update of the lateral inhibition model suggests that not transcription, but the activity of Dl is regulated in a feedback loop among the cells of the PNC [18]. In this model the activity of Notch results in activation of expression of the Bearded proteins, which in turn suppress the activity of Neur and therefore that of Dl. In this case, a differential activity of the Notch pathway should be observed. However this model is not in agreement with the finding that Neur is expressed only in the emerging SOP, but not in its neighbours, which are inhibited by it [12].

Most experiments that addressed the mechanism of SOP formation were performed more than a decade ago. In the meantime novel markers and mutants became available that allow a more precise look. We therefore re-examined the process. Our data suggest that the inhibitory Notch signal is restricted in range to the next cell. We found that a band-like region with changing proneural activity exists in the notum, whereby the peaks constituting the PNCs. The SOP appears to be chosen by an unknown mechanism among cells of a subgroup within the PNC that is defined through the requirement of the activity of Neur. We failed to find evidence for the described feedback loop of the lateral inhibition model. Rather, the Notch pathway has two functions: it provides a baseline of activity in the proneural band that defines the PNCs and the subgroup. It also mediates a strong inhibitory signal emitted by emerging SOP to inhibit the same fate in its neighbours. We found that Emc is required in all cells of the imaginal discs to suppress the proneural state. In the absence of *emc* function, the cells are in a proneural state from which several proceed to become SOPs, even in the absence of the function of *ac* and *sc*. These findings suggest that one important function of the proneural proteins is to neutralise Emc. Moreover, the Notch mediated selection of the SOP is independent of the proneural activity of Ac and Sc. The presented results indicate that the selection of the neural precursor in *Drosophila* is more similar to that in vertebrates than anticipated.

## Results

### Notch signalling during selection of the SOP

**Figure 1:**
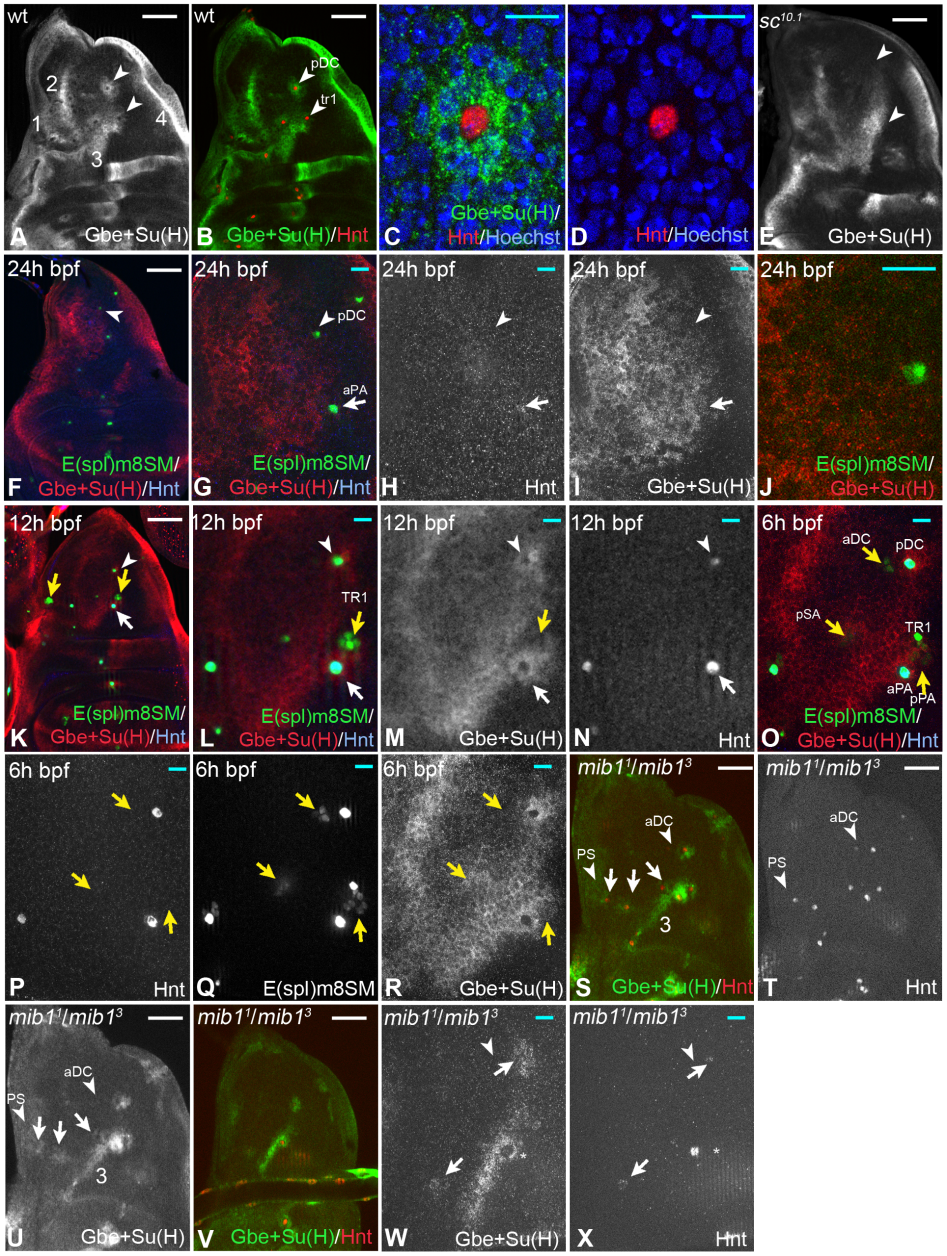
Expression of the Notch activity reporter Gbe+Su(H)-lacZ during SOP development in the notum. SOPs are labelled by Hnt expression. (A, B) Overview. The expression pattern contains 4 large stripe-like domains (1–4). The arrowheads highlight the halos around SOPs. (C, D) Magnification of the pDC region. The elevated expression of Gbe+Su(H) (halo) labels only the immediate neighbours of the SOP. (E) These halos are absent in *sc^10.1^* mutant discs (arrowheads highlight the positions of the halos also highlighted in (B)). (F–R) Expression of Gbe+Su(H), Hnt and E(spl)m8SM during selection of several SOPs. Staging is according the emergence of the SOPs determined by Huang et al. (1991). (F–J) A disc of the mid-third instar stage (24 h bpf). The arrow highlight the position of the aPA and the arrowhead that of the pDC. The aPA already weakly expresses Hnt, indicating that it is determined, whereas the pDC only expresses E(spl)m8SM. No differential staining of Gbe+Su(H) is observed. (K–N) A disc around 12 h bpf. The pDC and aPA SOPs expresses Hnt, indicating that they are determined. The halo is now clearly visible (arrow and arrowhead). The yellow arrows highlight the positions of the next arising SOPs. A small group of cells at the position of the future SOP express E(spl)m8SM, but not Hnt indicating the on-going selection. Similar groups of E(spl)m8SM expressing cells can also be observed at other position where SOPs are next selected, such as the aPA/tr1, pSA and aNP positions (yellow arrows in K–R). At all these positions no differential expression of Gbe+Su(H) can be observed. (O–R) A disc around 6 h bpf. Groups of E(spl)m8SM expressing cells can now be observed at the aDc and pSA positions where the next SOPs arise (yellow arrows). Again no differential expression of Gbe+Su(H) can be observed. (S–U) Expression of Gbe+Su(H) and Hnt in *mib1* mutant wing discs. (S, T) A late third instar disc where several SOPs are determined. The normal pattern of SOPs is recognisable and halos are observed at most SOP positions (arrows). The additional halos are recognisable due to the loss of several expression domains of Gbe+Su(H). Note, the remaining stripe 3 of the Gbe+Su(H) expression pattern (compare (U) with (A)). The arrowhead points to the region where the aDC and PS SOPs develop. No differential expression of Gbe+Su(H) can be observed. (V–X) An earlier disc where the nascent SOP at the pDC position just initiates expression of Hnt (arrow). It expresses Gbe+Su(H) just like its immediate neighbours. Later the expression is switched off in the SOP (see arrowhead in (A)). The arrowhead in (R, S) points to the aDC position where no Hnt expression is visible and the selection process is on-going. White scale bar 50µm; cyan scale bar 10µm.

In order to determine the activity of the Notch pathway during SOP selection, we performed several experiments. First, we monitored expression of the Notch activity reporter Gbe+Su(H) together with the SOP marker Hindsight (Hnt) in the notum of wing discs at the late third larval instar stage. Hnt is a marker for mature, already determined SOPs [19]. Expression of Gbe+Su(H) in the notum is complex and comprises many domains, which report the activation of Notch in several parallel running processes (Fig. 1A, B, [20]). Nevertheless, we observed an elevation of expression of Gbe+Su(H) (halo) in cells around several Hnt positive SOPs (Fig. 1A, B, arrowheads). We counted that this halo contained between 6 to 9 immediate neighbours (Fig. 1C, D). We found the same halos around mature SOPs using a recently available GFP variant of Gbe+Su(H), termed NRE-pGR [21]. In order to test whether the halos are related to SOP development, we monitored the expression of Gbe+Su(H) in *sc^10.1^* mutant imaginal discs, which lack the function of the proneural genes *ac* and *sc*, which are responsible for bristle formation in the notum. In these discs, the halos were absent, while all other domains of expression were unaffected (Fig. 1E, arrowheads, compare with A). Thus, the halos are caused by Ac/Sc dependent Notch signalling in PNCs. The observed halos suggest that the selected SOP sends a strong signal that activates the Notch pathway in its direct neighbours.

The lateral inhibition model predicts differential activity of Notch among cells of the PNC during SOP selection. This should be reflected in differential expression of Gbe+Su(H), with decreasing expression in the nascent SOP and increasing expression in its neighbours. To test this prediction, we looked at SOP formation at different times and at different positions. We used *E(spl)m8*-SM-GFP. In contrast to *E(spl)m8*, this construct is not responsive to Notch activity due to deletion of its Su(H) binding sites in its promoter [22]. It is the earliest marker for nascent SOPs and expressed well before Hnt [22], [23]. We found that this marker is initially expressed in a small group of cells in the PNC from which the SOP arises (Fig. 1 K-M, O-R, yellow arrows) and is then restricted to the emerging SOP before it expresses Hnt. We failed to observe any differential expression of Gbe+Su(H) around *E(spl)m8*-SM-GFP positive cells, before the onset of expression of Hnt. Expression was uniform and weak during the time the nascent SOP expresses only *E(spl)m8*-SM-GFP (Fig. 1F-R, arrow and arrowhead for the SOPs arising at the pDC and aPA positions). The halos formed at the time the SOP enlarged and initiates Hnt expression. These results suggest that activation of Notch appears to be uniform during the selection of the SOP. Only after its selection and maturation to the Hnt positive state, it sends an inhibitory signal to its neighbours via the Notch pathway.

Expression of Gbe+Su(H) in the notum is complex and includes four stripe-like domains (1-4 Fig. 1A), the halos and a diffuse weak expression between the domains (Fig. 1A). This complex pattern complicated the analysis at several SOP positions. To get rid of the irrelevant domains of expression of Gbe+Su(H), we performed the further analysis in *mib1* mutants. Loss of *mib1* function, which encodes the ubiquitously expressed E3-ligase required for DSL ligand activity, affects only a subset of Notch-dependent processes and MC formation is only mildly affected, resulting in the formation of a few supernumerary bristles [24], [25]. We found that SOPs emerged in the normal sequence, but earlier in *mib1* mutants. Ectopic SOPs emerged later. Moreover, the stripes of Gbe+Su(H) were lost or dramatically reduced with the exception of stripe 3 (see Fig. 1A, S, U). The halos around the SOPs remained intact and became recognisable at new positions (Fig. 1S, U, arrows). As in wildtype nota, the halos of Gbe+Su(H) expression were present only around mature, Hnt-positive SOPs. Also in this background, differential expression of Gbe+Su(H) during SOP formation of the aDC position was not observed (arrowhead in Fig. 1S-X, arrowhead). Note, expression of Gbe+Su(H) was observed also in the nascent SOPs at least at some positions, such as the PNP and pDC (arrows in Fig. 1W, X). We observed this also in wildtype discs, although not in that clarity because of the additional expression domains. This observation suggests that Notch is still active in the nascent SOP or the stable ß-galactosidase is not degraded at this time of SOP development. Expression of Gbe+Su(H) disappeared in fully determined SOPs, indicating that the Notch pathway is switched off and that differences of Notch activity among cells of the PNC can be detected (white arrow and arrowhead in Fig. 1M, asterisk in W). Altogether, the observations support the notion that a differential expression of Gbe+Su(H) and therefore differential activation among the cells of a PNC occurs only after selection of the SOP. Hence, lateral inhibition appears to occur after the SOP is selected, but not during its selection.

### Determining the range of the Notch signal

**Figure 2:**
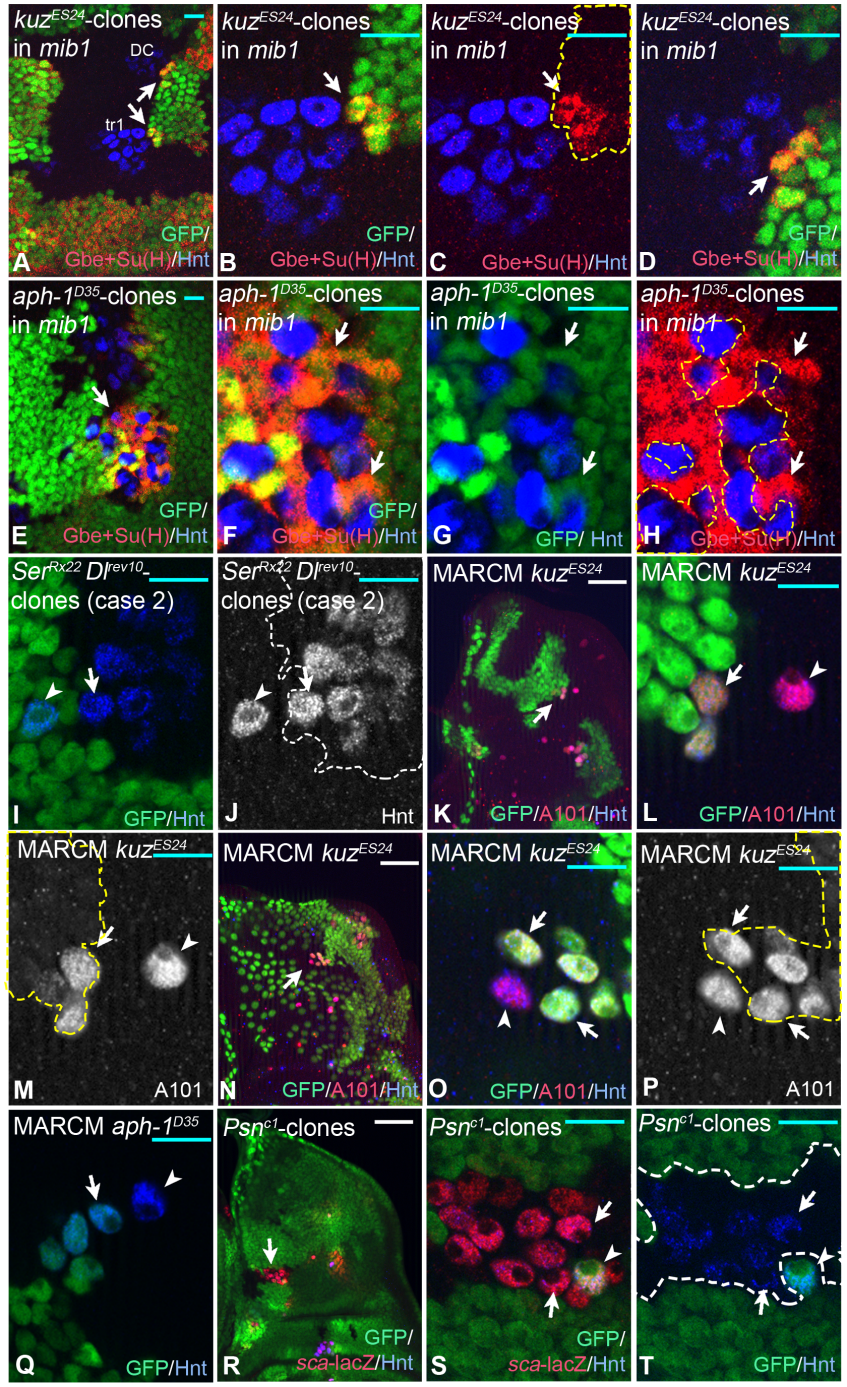
The range of the Notch signal. Clonal analysis of several neurgenic mutants. The clones are labelled either by absence or presence (MARCM) of the GFP marker and outlined by the dashed lines in (C, F, I, L, M, P). Clones are induced with hsFlp during the first instar stage. (A–D) A *kuz* clone in *mib1* mutant wing discs. Clones are labelled by the loss of GFP. (B–C) Magnifications of the regions highlighted in (A) with the lower arrow. Arrows in (B–D) highlight the regions where the mutant SOPs contact wildtype cells. Gbe+Su(H)-lacZ is elevated in the wildtype neighbours contacting mutant SOPs. (D) Magnification of the regions highlighted in (A) with the upper arrow. (E–H) An *aph-1* clone in *mib1* mutant wing discs labelled by the loss of GFP. (F–H) Magnifications of the regions highlighted in (E) with the arrow. The mutant SOPs induce Gbe+Su(H)-lacZ in the surrounding *mib1* mutant cells. (I, J) A *Ser Dl* double mutant clone (case1) labelled through the absence of GFP. The arrow points to the mutant SOP that developed one cell diameter away from the wildtype SOP (arrowhead). (G–P) Examples for case 2. (K–P) *kuz* MARCM clones in wildtype discs, positively labelled with GFP. The SOPs are marked by the A101-lacZ, a lacZ insertion in *neur*. (L, M) and (O, P) are magnifications of the regions highlighted with the arrows in (K) and (N) respectively. The arrowhead in (L, M) point to a wildtype SOP that is located one cell diameter away from a mutant SOP (arrow), the arrowhead in (O, P) to a wildtype cell that is in direct contact with mutant SOPs (arrows). In both cases the wildtype SOP cannot be inhibited from adopting the SOP fate by the mutant SOPs close by. (Q) A MARCM clone of *aph-1*. The arrowhead points to the wildtype SOP (arrowhead) located near a mutant one (arrow). As in the case of *kuz* clones, the wildtype SOP cannot be inhibited from adopting the SOP fate by the mutant SOPs close by. (R–T) A *Psn* mutant cell clone labelled through absence of GFP. The arrow in (R) highlights the region magnified in (S, T). The arrowhead in (S, T) points to the wildtype SOP that is surrounded by mutant ones (S, T, arrows). Also in this case, the wildtype SOP form in the presence of the surrounding mutant SOPs. White scale bar 50µm; cyan scale bar 10µm.

The observed halo of Gbe+Su(H) in cells around the SOP suggests that the inhibitory Notch signal emitted by the SOP reaches only adjacent cells. In order to experimentally confirm the short range of the signal, we generated *kuz* or *aph-1* mutant PNC cells by clonal analysis and tested their abilities to activate the Notch pathway in adjacent non-mutant cells (Fig. 2A-D). *kuz* encodes the ADAM protease that mediates the S2 cleavage of Notch to create the NEXT intermediate and *aph-1* encodes a subunit of the γ-secretase complex, which performs the subsequent S3 cleavage required to release NICD. Loss of function of each gene results in the inactivation of the Notch pathway at the signal receiving side. The lateral inhibition model predicts that these mutant cells should accumulate high proneural activity and become SOPs, because they cannot receive the inhibitory signal. Because of the high levels of proneural activity, they should express high levels of Dl and emit a strong Notch signal to their neighbours (S1A–C Fig.). To test this prediction, we monitored the expression of Gbe+Su(H) around mutant SOPs generated by clonal analysis. For simplification, we induced these clones in *mib1* mutant discs where several irrelevant domains of Gbe+Su(H) expression are absent. The function of *mib1* is not essential for SOP selection, since this process is largely mediated by the other E3 ligase Neur. We found that only *mib1* cells that were direct neighbours of the *mib1 kuz* or *mib1 aph-1* double mutant SOPs up-regulated Gbe+Su(H) expression (Fig. 2A–H, arrows). This observation suggests that the range of the Notch signal emitted by the *mib1 kuz* or *mib1 aph-1* mutant SOPs reaches only to their direct neighbours.

Next we tested over what distance the inhibitory Notch signal emitted by a SOP is effective in a functional assay. We analysed the ability of ectopic SOPs mutant for several neurogenic genes to influence the development of a wildtype SOP nearby. The lateral inhibition model predicts that:

1. cells of a PNC that are mutant for both ligands are unable to emit an inhibitory signal, but able to receive it. Therefore, these cells should never adopt the SOP fate if located adjacent to a strong signalling wildtype SOP (case 1, S1B, b Fig.).

2. ectopic SOPs mutant for genes that are required for signal-reception cannot be inhibited and should sent a potent inhibitory signal that should prevent all wildtype cells in its neighbourhood from adopting the SOP fate. Hence, no wildtype SOP should develop adjacent to a mutant SOP (case 2, S1C, c Fig.).

If the inhibitory Notch signal reaches a longer distance, e.g. through filopodia, the described effects should extend also to cells located farer away from the mutant cells (see also S1 b, c Fig.). We found that *Dl/Ser* double mutant cells (Fig. 2I, J, arrowhead), which are not able to inhibit their neighbours (case 2), adopted the SOP fate despite the presence of a wildtype SOP one cell diameter away (Fig. 2I, J, arrow). This suggests that the wildtype SOP can only suppress the SOP fate in cells that are in direct contact to it.

SOPs mutant for *kuz* and *Psn* (Fig. 2K–T, arrow) could not prevent wildtype cells (Fig. 2K–T, arrowhead) from adopting the SOP fate at a distance of one or more cell diameters away (case 2, Fig. 2K–M). Moreover, we found several cases where these mutant SOPs are unable to inhibit a wildtype SOP in direct contact to them (Fig. 2N–T, arrowhead). This suggests that cells at the positions where the SOPs normally arise, can adopt the SOP fate, even next to strongly signalling cells. Cells at these positions appear to be pre-selected to become SOPs and immune to the inhibitory signal. Altogether, the results strongly support the conclusion that the effective inhibitory Notch signal emitted by the nascent SOP reaches only its direct neighbours and that the SOP in the PNC is pre-selected. We have previously arrived to the same conclusions upon clonal analysis of *Su(H)* mutants [19]. A problem with this analysis was that *Su(H)* mutant cells fail to properly express Neur and several target genes of the Notch pathway are de-repressed in them [19], [26]. This might cause an abnormal behaviour of the mutant cells during SOP selection. All these effects are absent in the *kuz* and *Psn* mutant cells analysed here.

### Expression of Dl and Ser

We wondered how each ligand contributes to the activation of the Notch pathway in the notum. To answer this question, we compared the expression of Ser and Dl relative to that of Gbe+Su(H). We found that the combined expression of both ligands together covers most of the notum (S2A–G Fig.). The domains of high Dl expression are shifted with respect to that of Gbe+Su(H) expression (S2A, B, E Fig.). Thus, strong expression of Dl does not correlate with high Notch activation. To investigate the expression of Dl and Ser in the PNC, we used *sca*-lacZ, a faithful marker for proneural activity. In regions of the PNCs, Dl appears to be uniformly expressed (S2H, I Fig., arrow and arrowhead). In the region of the aPA/Tr1 cluster expression of Dl was significantly lower (S2B, E Fig., arrow). The domains of high expression of Ser correlated better with that of high Notch activity, although it was not a perfect match (S2A, C, F Fig.). Consequently, the domains of high expression of Dl and Ser are overlapping (S2D–F Fig.).

Optical sections showed that all cells of a PNC contain Dl positive vesicles (S2H, I Fig.). Most of these vesicles (89%, n =  276) were Rab7 positive and therefore maturing endosomes ([27], S2H, I Fig.). We did not observed differences in Dl expression or distribution among cells of the PNCs (S2H, I Fig.).

Expression of Dl was unchanged in *sc^10.1^* mutants (S2J Fig., compare with E), suggesting that the proneural factors do not regulate the expression of Dl during the determination of the SOP of the MCs. This is in agreement with the analysis of expression of Dl during mcs development and our observation that the expression of Gbe+Su(H)-lacZ is unaffected, with the exception of the lost halos ([28], Fig. 1E).

### The notum contains a band-shaped region of proneural activity

**Figure 3:**
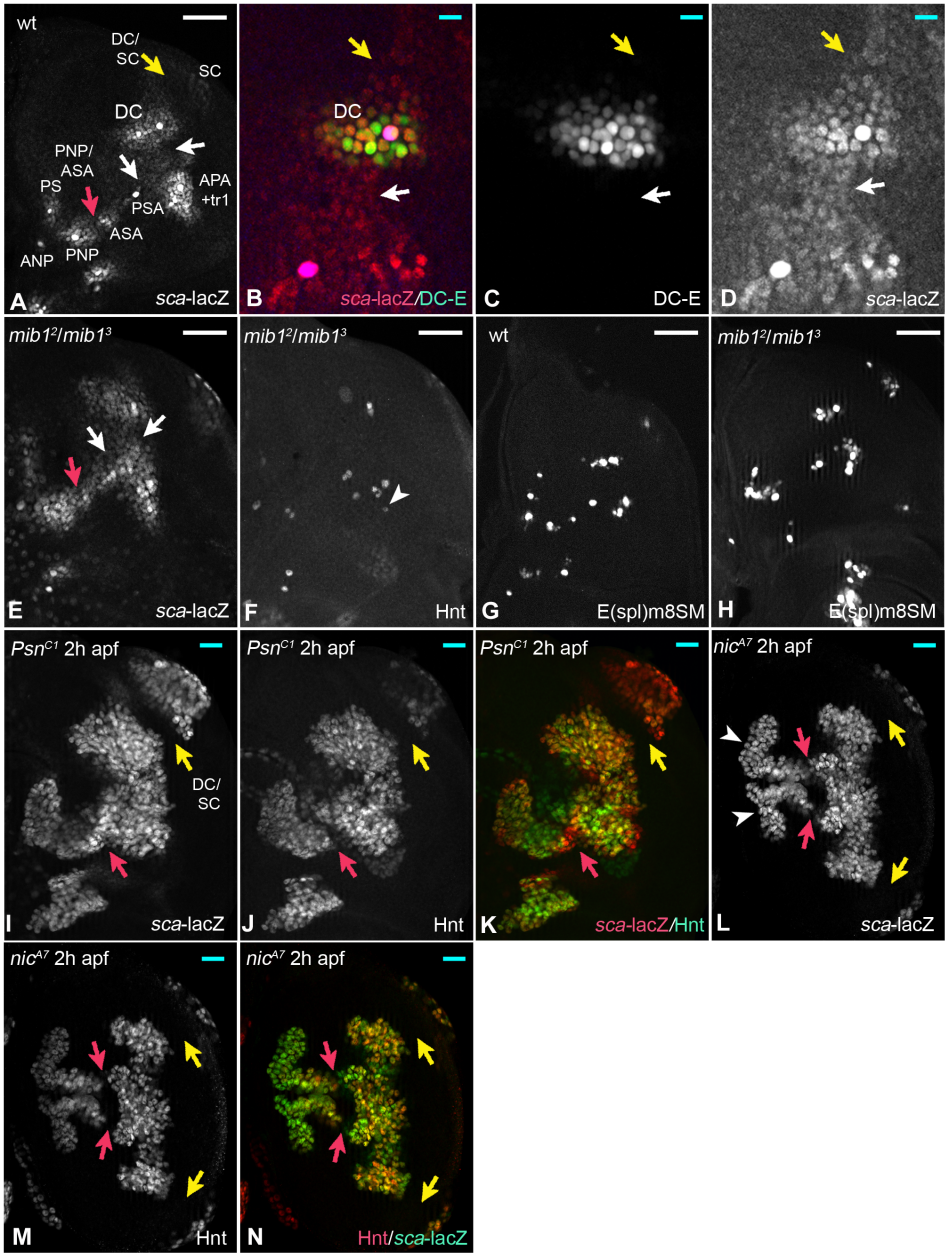
Loss of Notch pathway activity reveals the existence of a proneural band in the notum. The red and yellow arrows in various panels point to regions (PNP/ASA and DC/SC) where the cells adopted the SOP fate latest. (A) Expression pattern of *sca*-lacZ in the notum of a late third instar wing disc. The arrows point to the region with low expression between the PNCs. (B–D) The comparison of *sca*-lacZ with the expression of the DC-E confirmed that *sca* is expressed also between PNCs (arrows). (E) Expression of *sca*-lacZ is elevated between PNCs in *mib1* mutant disc causing some PNCs to fuse (arrows, compare with (A)). (F) Pattern of the SOPs in the disc shown in (E). The arrowhead highlights an ectopic SOP. (G, H) Expression of the early SOP marker E(spl)m8SM in wt (G) and *mib1* mutant discs (H). (I–K) In the absence of *Psn* function, the regions between the PNC up-regulate the expression of *sca*-lacZ and eventually form of a band-like region at 2 h apf. The vast majority express Hnt and *sca*-lacZ, indicating that they became SOPs. (L-N) The same is observed in *nic* mutant wing discs. The duplicated white and yellow arrows in (L) highlight the duplication of the proneural band caused by a wing to notum transformation often observed in these mutants. White scale bar 50µm; cyan scale bar: 10µm.

We previously noticed that *sca*-lacZ, which faithfully reports proneural activity, is weakly expressed also in regions between PNCs [23]. This is highlighted by the comparison of *sca* with the expression of the general enhancer that regulates the expression of *ac* and *sc* in the DC cluster (DC–E) ([29], [30], Fig. 3A–D). The observation suggests the existence of a corresponding band-shaped curved region of changing proneural activity (proneural band), with the PNCs being only peaks of this region. We found that expression of *sca* is elevated in regions between PNCs in *mib1* mutant nota (Fig. 3A, E, arrows). Moreover, more cells expressed the earliest SOP marker *E(spl)m8*-SM-GFP (Fig. 3F–H). We quantified this for the region of the aPA1/tr and pSA clusters and found that in wt 6 ± 2 cells are positive for *E(spl)m8*-SM-GFP (n =  6), while 18 ± 4 are positive in *mib1* mutants (n =  8). Thus, more cells accumulate high proneural capacity, which is actively suppressed during normal development through the activity of the Notch pathway. We were curious whether the cells of the proneural band could be coaxed to adopt the SOP fate if the activity of Notch is abolished. Indeed, this was the case for most of the cells of the band: In *Psn* or *nic* mutant nota, where the γ-secretase complex is inactive, the PNCs increased in size over time and eventually fused at 2h after puparium formation (apf) to form a continuous band of strong *sca* expression (Fig. 3I–N). Moreover, most cells eventually expressed Hnt, indicating that they adopted the SOP fate (Fig. 3I–N). There are two exceptions: cells located between the DC and SC clusters never became SOPs, although they expressed *sca* (yellow arrow in Fig. 3I–N). Cells in the region between the PNP and ASA clusters expressed high levels of *sca*, but only few had adopted the SOP fate (red arrow in Fig. 3I–N).

Altogether, these results reveal the existence of a proneural band in the notal region of the wing disc, which is divided in regions of high and low proneural activity. They uncover a novel role of the Notch pathway, which is the definition of the PNCs through suppression of the proneural activity in cells located between the clusters. This activity is only partly generated through Mib1, since the cells between the PNCs do not adopt the SOP fate in its absence, although they have increased levels of proneural activity.

### Loss of *neur* function reveals a subgroup in the PNC

**Figure 4:**
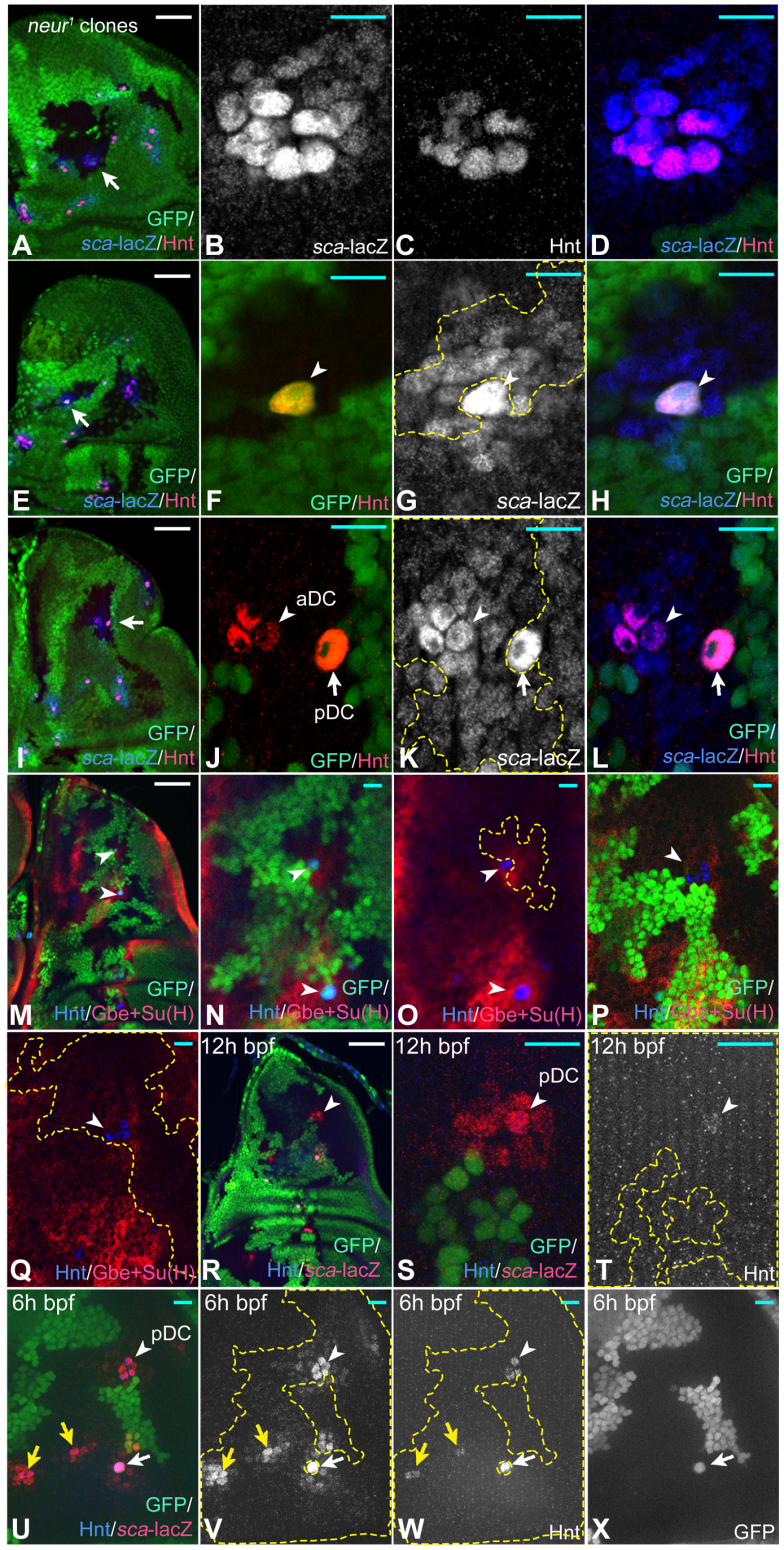
Clonal analysis of *neur*. Clones are labelled by the loss of GFP (green). (A–D) A *neur* mutant clone that includes the complete aSA cluster (arrow in A). The region is shown at higher magnification in (B–D). Comparison of *sca* and Hnt expression reveals that a subgroup of 7 cells has adopted the SOP fate in the absence of *neur* function. (E–H) A clone where a wildtype SOP is surrounded by *neur* mutant cells (arrow in E). (F–H) Magnification of the region highlighted by the arrow in (E). Only the wildtype cell has adopted the SOP fate indicated by Hnt expression (arrowhead in F, G, H). Hence, the wildtype Neur positive SOP can prevent all *neur* mutant neighbours from becoming SOPs. (I) A clone that includes the DC cluster (arrow). This region is shown at higher magnification in (J–L). At the aDC position all cells are mutant and three cells have adopted the SOP fate as indicated by expression of Hnt (arrowhead). At the pDC position only the GFP positive wildtype cell expresses Hnt (arrow). Note, while this SOP inhibits its neighbours, it is unable to prevent the SOP fate in the mutant cells only two diameters away (arrowhead). This indicates that the Neur dependent signal emitted by the pDC SOP reaches only the adjacent cells. (M–O) In cases where the clones include a wildtype SOP, a halo of Gbe+Su(H) expression is observed. Arrowheads in (M) point to the regions shown in (N, O) at higher magnification. Arrowheads in (N, O) point to the wildtype SOPs surrounded by the *neur* mutant cells. (P, Q) The halos were lost in cases where the whole cluster is mutant (arrowhead). (R–X) Expression of Hnt and *sca*-lacZ *neur* mutant PNCs at 12 h and 6 h bpf. At the position highlighted by the arrowhead, four cells express Hnt, but one stronger than its neighbours. The cell is also larger. Only one cell expresses Hnt at the other position of the mutant cluster (arrow). (T–W) A *neur* clone that includes the DC cluster at 12 h bpf. The arrowhead highlights the position of the pDC in the early arising DC cluster, the yellow arrows that of later arising PNCs. The white arrow highlights a single wildtypic SOP surrounded by mutant cells. (S, T) Magnification of the DC region highlighted by the arrowhead in (R). At this early time the pDC arises. Although all cell of the cluster are *neur* mutant, only one cell has elevated levels of *sca* expression (S) and weakly expresses Hnt (T) at this stage. This indicates that this cell has elevated proneural activity and is already determined as SOP. Thus, this cell is advanced in its development towards the SOP fate. In the older disc shown in (U–X) four Hnt expressing SOPs can be seen at the pDC position. This indicates that the advanced cell was not able to inhibit its neighbours in the absence of Neur function. The yellow arrows point to the late arising cluster mutant for neur. In these cluster only one Hnt expressing cells is recognised, indicating the these cells are also advanced in their development. Note, that the expression of *sca*-lacZ is not elevated between the PNCs in the mutant territory as observed upon complete loss of Notch function. White scale bar 50µm; cyan scale bar: 10µm.

In the imaginal discs Neur function is restricted to neural development and the neurogenic phenotype of *neur* mutants is milder than that of mutants of other neurogenic genes [9]. We therefore determined how many cells of a PNC adopt the SOP fate in the absence of *neur* function. To do so, we compared expression of *sca* and Hnt in *neur* clones, induced by *ptc*Gal4 UAS *Flp*. It revealed that only a subgroup of the PNC (*neur*-group) adopted the SOP fate (Fig. 4A–D). The *neur*-group comprised up to eight SOPs, which is approximately the sum of the SOP plus its neighbours (see Fig. 1C) and is in agreement with the results of a previous study [9]. Importantly, we observed many cases where one or a few cells in the *neur*-group were wildtype. In these cases, only one SOP formed (Fig. 4E–H, U-X). This SOP was always a wildtype cell, indicating that a Neur positive cell can efficiently inhibit the other cells of the subgroup (Fig. 4E–H, arrowhead, U–X, white arrow). A nice example including both described situations in the DC cluster is depicted in (Fig. 4I–L, arrowhead and arrow). In cases where the SOP was wildtype, we observed a halo of Gbe+Su(H) expression in the adjacent cells, confirming that it can send a potent inhibitory Notch signal to its neighbours in the presence of Neur (Fig. 4M–O, arrowheads). If all cells of the subgroup were mutant, a clear halo was absent (Fig. 4P, Q, arrowhead). Altogether, the results indicate that a subgroup exists within the PNC (*neur*-group), which requires the activity of Neur in at least one cell to prevent the formation of supernumerary SOPs through lateral inhibition.

The SOPs in *neur* mutant territories appear to emerge in a sequence. This is indicated that only one SOP can be detected with Hnt or elevated *sca*-lacZ expression during early phases of mutant PNCs development (see Fig. 4R-X, arrowhead for the early arising pDC, yellow arrows for the later arising PNCs). These observations support the notion that one cell of the *neur*-group is ahead in its development to become a SOP. During further development more cells become Hnt positive SOPs in mutant PNCs indicating that the advanced cell is unable to inhibit its neighbours in the absence of Neur (Fig. 4U–X, arrowhead, DC cluster).

In order to investigate how the *neur* group might be defined, we monitored the expression of DC-E. This enhancer controls the general expression of Ac and Sc in the DC cluster, but not their elevation in the nascent SOP (S3 Fig., [30]). To identify the position of the future SOP, we used the elevation of *sca*-lacZ expression in one cell of the PNC, which is the first known sign of SOP formation [31]. We found that the expression of the DC-E is not uniform and a group of cells at the position where the SOP arises expressed higher levels. This is exemplified for the two SOPs of the DC cluster (S3 Fig., arrowheads). Thus, differential expression of Ac and Sc from the beginning of the PNC is likely to contribute to the determination of a subgroup of cells from which the SOP arises. This subgroup is probably the *neur* group. If the cells of the *neur* group possess more proneural activity, they should adopt the SOP fate before the rest of the PNC in the absence of Notch activity. Hence, one should initially observe small groups of SOPs in *Psn* mutant discs. The groups should increase in size during the third instar. This is what we observed (S4 Fig.). Note, that during the third instar new SOPs are added at the edges of the existing group. Thus, the cells of the PNC have different proneural activity with the subgroup possessing the highest proneural activity, allowing them to adopt the SOP fate faster than the rest of the PNC. This group probably requires higher Notch activity to suppress the SOP fate provided by Neur. Outside the subgroup the proneural activity decreases towards the edges of the PNC.

In contrast to complete loss of function of Notch, cells of the PNCs outside the *neur* group and of regions between the PNCs never became SOPs upon loss of *neur* function. Moreover, no elevation of *sca*-lacZ expression in *neur* mutant cells of the proneural band outside the *neur* subgroup was observed (Fig. 4U,V, compare with Fig. 3A). This observation suggests that the requirement for function of Neur is restricted to the subgroup.

### Emc is required to suppress proneural activity in imaginal disc cells

**Figure 5:**
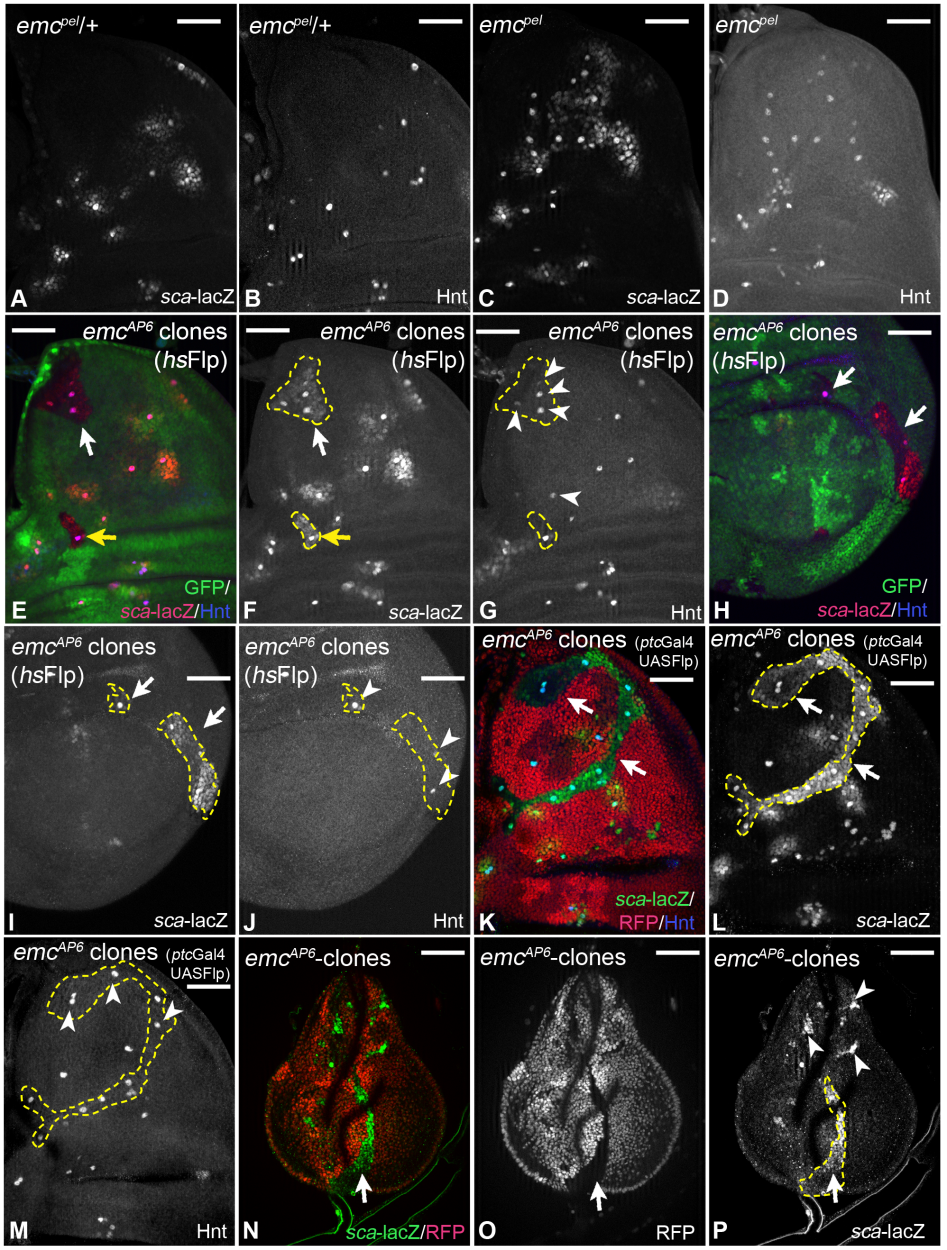
Analysis of *emc* function. (A–D) *emc^pel^* mutant phenotype. (A, B) Expression of *sca* and Hnt in a heterozygous control disc. (C, D) Expression of the marker in an *emc^pel^* homozygous disc. The expression of *sca* has expanded and many more cells express Hnt in the mutant disc, indicating an expansion of the proneural band and formation of more SOPs at ectopic positions. (E–P) Clonal analysis of *emc*. Clones are labelled by the loss of GFP and outlined in several panels. (E–J) hsFlp induced *emc* null mutant clones. (K–M) Null mutant clones induced by UAS *Flp* driven by *ptc*Gal4. All cells of the clones express elevated levels of *sca* (arrows) and several also Hnt (arrowheads). Note, that elevated *sca* expression and SOP formation is also observed in the posterior compartment (H–I, arrows, arrowheads in J). (N–P) Ectopic *sca* expression is already observed in early third larval instar discs long before the normal PNCs appear (arrows and arrowheads). White scale bar 50µm.

In wing imaginal discs homozygous for the weak allele *emc^pel^*, ectopic SOPs arise in regions outside the proneural band and expression of *sca*-lacZ is expanded ([14]; Fig. 5A–D). These observations indicate that Emc is involved in defining the proneural band. We went on to test the effects of two null alleles, *emc^1^* and *emc^AP6^*, which have been recently used for clonal analysis in combination with the *Minute* technique [5]. We were able to recover *emc* clones with both alleles in imaginal discs even without the *Minute* technique (Fig. 5E–J). We obtained the largest clones using a *ptc*Gal4 driven UAS *Flp* construct (Fig. 5K–M). Independently of the technique, we observed that *emc* mutant cells activated ectopic *sca*-lacZ expression (Fig. 5E–M). Moreover, several mutant cells developed further to become ectopic SOPs (Fig. 5E–M). These results indicate that *emc* mutant cells adopt a proneural state from which they can progress to become SOPs. Note, that loss of *emc* function caused these effects also in cells of the posterior compartment where *ac* and *sc* are not expressed (Fig. 5H–J). Interestingly, we found that mutant SOPs tended to arise away from the clone boundary.

We observed elevation of *sca* expression already in clones of early third instar discs (Fig. 5N–P). Thus, adopting a proneural state is an immediate response of the cells to loss of *emc* function. The same response to loss of *emc* function was found in the leg disc. Hence, it appears to be a general response to loss of *emc* activity in imaginal disc cells. Emc inhibits only the activity of proneural bHLH proteins, but not their expression. Therefore, the cells of the imaginal discs must posses a low level of proneural proteins, whose activity become elevated upon loss of Emc.

### The Notch pathway mediates selection of the ectopic SOPs in *emc* mutant territories

Only a fraction of cells in the *emc* mutant clones developed to ectopic SOPs, although all cells elevated their proneural activity (4, 4 ± 1,8% in *emc* null mutant clones containing at least 100 cells, n =  6). These SOPs were well separated from each other, suggesting that the Notch mediated selection process operates in *emc* mutant territories. To test this notion, we first monitored the expression of Gbe+Su(H) in *emc* cell clones. We found that it was not affected (S5A–D Fig.). We could detect halos Gbe+Su(H) expression around ectopic Hnt positive SOPs, indicating that they send a strong inhibitory signal to their neighbours (S5A–D Fig., arrows). Expression of Dl was unaffected (S5E–K Fig., arrow and arrowhead).

**Figure 6:**
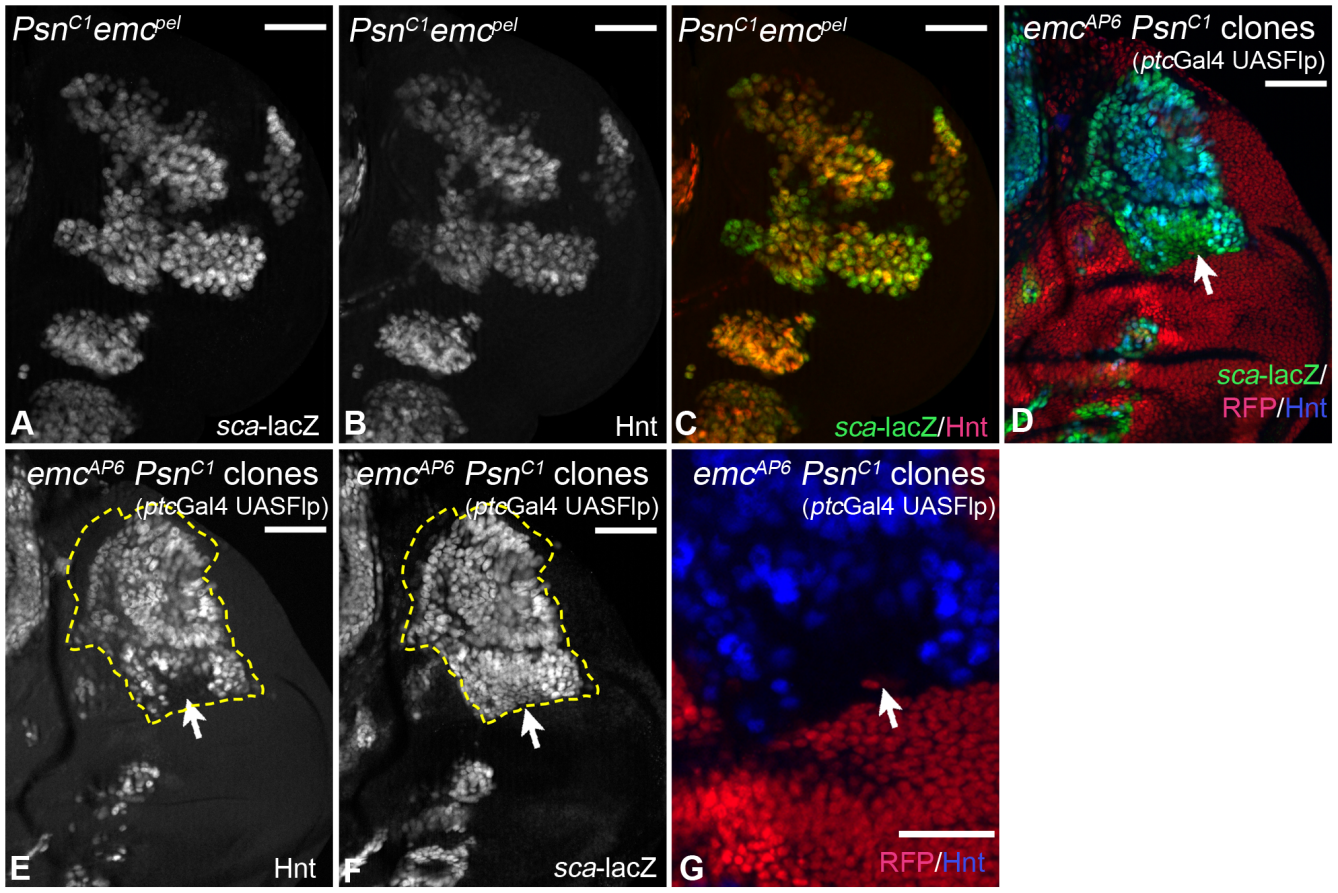
Consequences of loss of *Notch* pathway activity in *emc* mutant cells. (A–C) In *Psn^C1^ emc^pel^* double mutant discs, expression of *sca* is stronger and expands more than in *emc^pel^* mutants. Moreover, most *sca* expressing cells express Hnt, indicating that they became SOPs (compare with Fig. 5C, D). (D–G) Analysis of null mutant *emc Psn* clones. Clones are labelled by the loss of RFP and outlined in some panels. A large clone in the notum is highlighted by the dashed line. The expression of Hnt in addition to *sca*-lacZ indicates that most cells of the clone became SOPs, with the exception of the region in the hinge highlighted with the arrow. The arrowhead points to the small anterior region, which is devoid of *sca*-lacZ and Hnt expression. (G) Magnification of the notal area highlighted with the arrow in (D–F). White scale bar 50µm; cyan scale bar: 10µm.

We went on to investigate the consequences of loss of Notch function in *emc* mutant wing discs in two ways: We analysed flies homozygous for the hypomorphic allele *emc^pel^* and found that, in contrast to *emc^pel^* single mutants, all *sca*-lacZ positive cells became Hnt positive SOPs in *emc^pel^ Psn^c1^* double mutants, with exception of very few cells at the edges, which expressed *sca* only weakly (Fig. 6A, C, arrows). We further analysed the consequences of inactivation of the Notch pathway in *emc* null mutant cells (*emc^AP6^ Psn^c1^* clones). We found that most of the *emc^AP6^ Psn^c1^* mutant cells that express *sca*-lacZ adopted the SOP fate in the absence of Notch activity (80,5 ± 16% in clones containing at least 100 cells, instead of 4,4 ± 1,8% in *emc* null mutant clones, n =  6), with the exception of cells in the central part of the hinge region, which did not progress beyond the *sca* expressing proneural state until the stage of analysis (highlighted by the arrow in Fig. 6D–G). In addition we found that an anterior small stripe of the notum fails to elevate *sca*-lacZ and Hnt expression (Fig. 6D–G, arrowhead). This indicates that these cells are not capable to become SOPs. In summary, the analysis showed that the activity of the Notch pathway is required for the selection of SOPs in *emc* mutant territories.

### The activity of Ac and Sc is dispensable for bristle development in the absence of *emc* function

**Figure 7:**
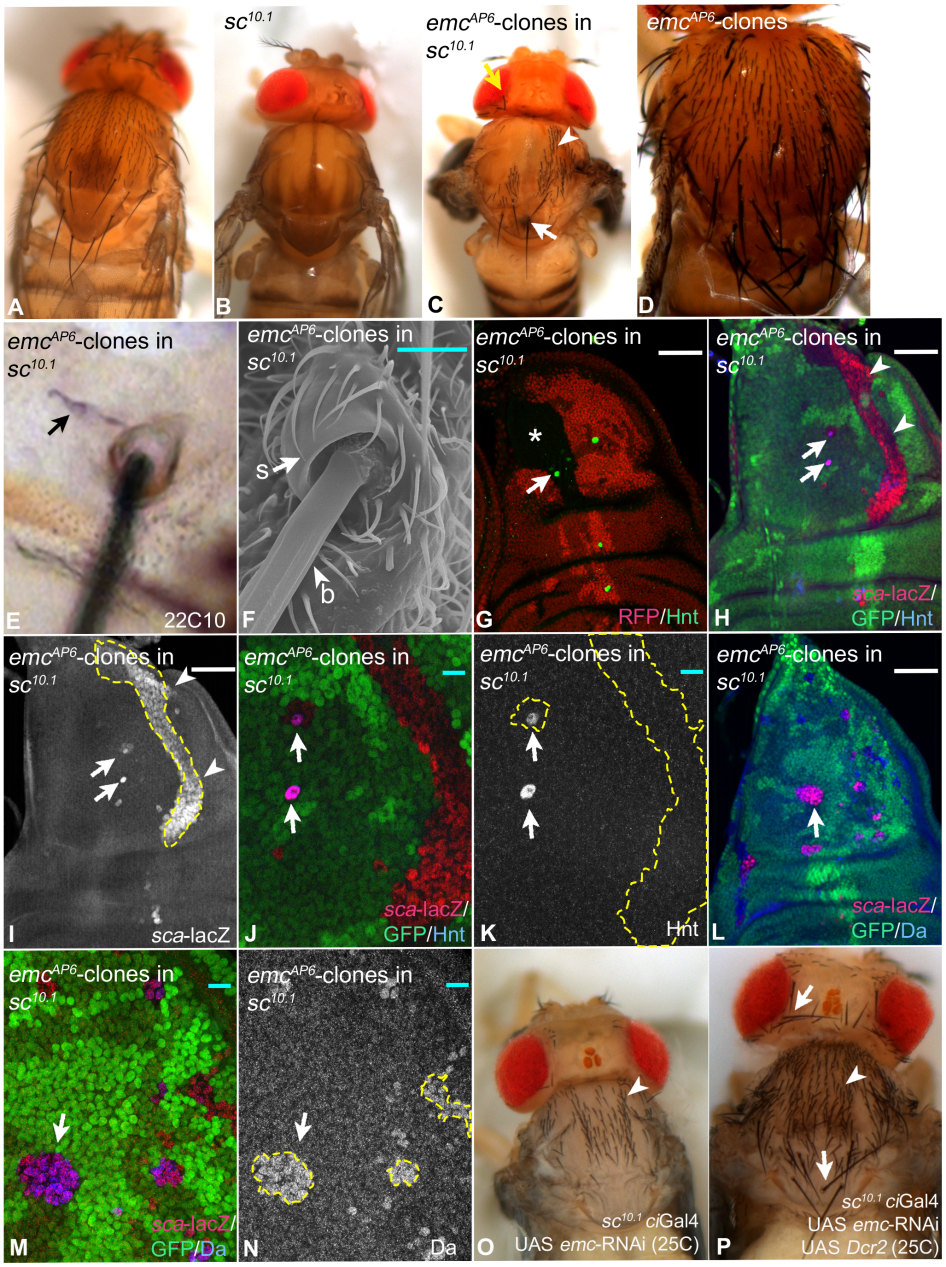
Loss of *emc* function causes the re-appearance of Mcs and mcs in *ac sc* double mutant flies. (A–C) Loss of *emc* function in *sc^10.1^* mutant flies. (A, B) Notum of wildtype and *sc^10.1^* mutant flies respectively. (C) Notum of a *sc^10.1^* mutant fly bearing *emc*-clones induced with *ptc*Gal4 UAS *Flp*. MCs and mcs re-appear in patches (arrow and arrowheads respectively). Note, that the regional distribution of the two types of bristles is similar to that in the wildtype with MCs at the edges and mcs in the centre. (D) Wildtypic notum bearing *emc* clones exhibit supernumerary and ectopic MCs. (E) The *sc^10.1^ emc* mutant bristles possess 22c10 positive neurons (arrow). (F) The REM analysis shows that they also possess normal socket and bristle cells. (G–K) Emergence of SOPs in *emc sc^10.1^* mutant clones revealed by Hnt and *sca*-lacZ expression. The clones are outlined in some panels. (G) Several SOPs have formed in the clone area (arrow). The asterisk highlights the central regions of the future notum located in the clone. The later developing SOPs of mcs form in this region. (H–K) All *emc sc^10.1^* mutant cells elevate expression of *sca* (arrowheads) and some of these cells become SOPs (arrows). (J, K) Magnification of the area highlighted in (H, I) with the arrows and arrowheads. (L–N) The loss of *emc* function in *sc^10.1^* discs results in the elevation of Da expression (arrow in M, N). The arrow in (L) point to the region magnified in (M, N). (O, P) Expression of UAS *emc-RNAi* in *sc^10.1^* flies at 25C. (O) It causes the re-appearance of mcs (arrowheads). (P) Co-expression with Dcr2 causes the re-appearance of MCs (arrows) in addition to mcs (arrowheads). White scale bar 50µm; cyan scale bar: 10µm.

Loss of *emc* function causes SOP development in the posterior compartment of the wing disc, although Ac and Sc are not expressed there in wildtype discs. This raised the possibility that they are dispensable for SOP formation in *emc* mutant territories. To test this possibility, the consequence of loss of *emc* function in *sc^10.1^* flies was analysed [32]. *sc^10.1^* flies lack the function of *ac* and *sc* and consequently lack all bristles in the head and notum (Fig. 7A, B). We found that induction of *emc* null mutant clones in *sc^10.1^* flies resulted in the re-appearance of MCs as well as mcs in both regions (Fig. 7C). Thus, the activity of Ac and Sc is dispensable for bristle development in the absence of *emc* function. This result also indicates that Emc regulates the formation of mcs. This involvement is only revealed in the *sc^10.1^* background, since most ectopic bristles induced by loss of *emc* function in a wildtype background are MCs (Fig. 7D). Note, that the bristles present in *sc^10.1^ emc* double mutant territories are well separated from each other, suggesting that the Notch mediated selection process operates despite the lack of the proneural genes.

We found that the *emc^AP6^ sc^10.1^* mutant bristles are associated with a 22C10 positive neuron and possess normal looking socket and bristle cells (Fig. 7E, F). Thus, the corresponding SOPs initiated the normal lineage. Although we observed bristles at ectopic positions in the *sc^10.1^* background, the distribution of MCs and mcs was not random. The MCs tended to develop in lateral and scutellar regions of the notum and the mcs developed in central regions, like it is observed in the wildtype situation. This indicates that positional cues exist in the absence of Ac, Sc and Emc, which contribute to the regional specification of bristles. However, the positioning of individual MCs was lost. For example the MCs highlighted with white arrows in (Fig. 7C and P) arose in a region of the scutellum normally devoid of bristles.

Analysis of corresponding double mutant wing imaginal discs revealed that expression of *sca* is elevated to similar levels as seen in *emc* clones (Fig. 7H–K). This indicates that *emc^AP6^ sc^10.1^* cells are in a proneural state despite the absence of the tissue specific proneural factors. Moreover, single cells progress to the SOP stage (Fig. 7G–K, arrows). Note, that the regions of clones that covered central areas of the notum did not form SOPs at this stage (see Fig. 7G, asterisk). Since we observe that these central areas are covered with the later developing mcs in the adult flies, we believe that the corresponding SOPs arise later. The double mutant cells also dramatically increased expression of Da (Fig. 7L–N, arrow). Thus, the previously observed elevation of Da expression in *emc* clones is independent of Ac and Sc [5]. In order to confirm that the loss of *emc* function is responsible for the re-appearance of the bristles and not a second mutation on the used chromosome, we depleted *sc^10.1^* mutant nota of *emc* function by expressing an UAS *emc*-RNAi constructs with *ci*Gal4, which drives expression throughout the anterior compartment. We observed the re-appearance of many mcs (Fig. 7O, arrowheads). To achieve maximal efficiency of depletion, we next co-expressed UAS *emc*-RNAi with UAS Dcr2. This resulted in the re-appearance of a higher number of mcs and also MCs (Fig. 7P, arrowhead and arrows respectively). These results confirm that the loss of *emc* function causes the re-appearance of bristles in *sc^10.1^* mutant nota.

### Development of proneural activity relative to expression of Emc

Recently a protein trap, Emc*-*YFP, which encodes a fully functional Emc-YFP fusion protein became available [33]. This was used to re-examine the expression of Emc during bristle development. The comparison revealed that the expression patterns of the previously available *emc*-lacZ and Emc-YFP are similar in the wing imaginal disc. However, *emc*-lacZ expression was elevated in determined SOPs of late third instar discs, while Emc-YFP was decreased and eventually switched off (S6A Fig.). Moreover, the “valleys” of expression of Emc-YFP were broader. This difference can be explained by the known stability of ß-galactosidase in *emc*-lacZ. The strong perdurance of ß-galactosidase in progenies of the expressing cells is likely to cause the observed gradual difference among cells around the SOP not seen with *emc*-YFP.

We compared the emergence of SOPs relative to Emc-YFP and *sca* (S6B-h Fig.). In early third instar discs the expression of *sca*-lacZ is initiated weakly throughout most of the notum (S6C, D Fig., asterisk). Within this domain of uniform expression, the PNCs arise in the previously described sequence and initially comprise few cells (S6D–F Fig.). Note, that at this time one cell eventually expresses higher levels of *sca* indicating that is has been pre-selected to become the SOP (arrowhead in S6F′′ Fig.). No Hnt expression was observed at this stage. Thus, the selection of at least the early arising SOPs occurs at a stage where the clusters comprise few cells.

As previously reported, Emc is expressed in all cells of the imaginal discs. In early third instar discs two domains of higher expression can be observed: one large central anterior located domain (domain 1) and a smaller posterior distal one (domain 2; S6C Fig.). An even smaller third domain (domain 3) follows at more anterior distal position (S6D Fig.). In late instar discs 5 domains of higher expression can be observed (S6H Fig.).

In general we found that the expression of Emc is low in the regions of the PNCs (high *sca* expression). For example, the Tr1 arises between domain 1, 2, and 3 (S6E-e Fig., arrow and arrowhead). Moreover, the early clusters arise at the edge of the large domain 1 in a band of low Emc expression. This band probably defines the proneural band.

We failed to find differential expression suggestive for a role of Emc to pre-select a cell within the PNC. The earliest known sign of SOP determination in a cell is the elevation of *sca*-lacZ expression. Upon close examination of Emc-YFP expression at this time at several positions, we failed to observe a clear reverse correlation between Emc-YFP and *sca*-lacZ expression (S6e, g Fig.). However, when the SOP is determined and expresses Hnt, the expression of Emc disappears (S6H, h Fig.).

We confirm the previous finding that the expression of Emc is unaffected in *sc^10.1^* mutants, indicating that it is independent of the proneural factors ([14]; S7A Fig.) However, we found very weak residual expression of *sca*-lacZ in the notum (S7A Fig., arrowheads). This hints to the presence of residual proneural activity. In order to test this possibility, we abolished the function of the Notch pathway in *sc^10.1^* discs (*sc^10.1^ Psn^C1^* mutants). The concomitant loss of Notch activity should lead to an increase of the proneural activity and, thus, in an increase of the residual expression of *sca*-lacZ. In agreement with this conclusion the *sca*-expression was increased in the remaining *ac* and *sc* independent PNCs of the radius of *sc^10.1^ Psn^C1^* wing discs that give rise to non-bristle type sensilla (arrows in S7B Fig.). The cells of these remaining clusters were also Hnt positive, indicating that they have adopted the SOP fate. However, we failed to observe an increase of the residual weak expression of *sca-lacZ* in the notum where the *ac sc* dependent PNCs are located (S7B Fig., arrow). This strongly suggests that the very weak residual expression of *sca*-lacZ in the notum is independent of proneural activity and that proneural activity is abolished in *sc^10.1^* nota.

In contrast to proneural genes, depletion of *da* function results in the loss of expression of Emc ([5], S7C Fig., arrow). This confirms previous findings that Da acts independently of the proneural genes to regulate the expression of Emc [5].

The analysis of expression of Emc-YFP in *Psn* mutants revealed that the regions where the cells of the proneural band are most resistant to become SOPs upon loss of Notch activity, e. g. between the aSA and pSA or the DC and SC clusters, are regions of high Emc expression (S7D Fig., red and yellow arrows). The overall expression of Emc appears not to be affected upon loss of *Psn* function. Altogether, these results suggest that Emc is involved in definition and subdivision of the proneural band, but does not pre-select the SOP in the PNCs.

## Discussion

In this study, we re-examined the development of the SOP of the MC using recently available reagents. We found evidence that strongly suggests that the range of the Notch signal is restricted to the next cell: The elevated expression of Gbe+Su(H) around the SOP is observed only in adjacent cells. In addition, cells of PNCs that are not able to receive the Notch signal, but can send a strong signal to adjacent wildtype cells, cannot prevent a wildtype cell from adopting the SOP fate at a distance of two cell diameters away. Likewise, cells that are not able to send a signal cannot be prevented by wildtype SOPs from adopting the SOP fate more than one cell diameter away. These results suggest that the discovered filopodia of the SOP, which contact more remotely located cells do not extend the range of the inhibitory signal to these cells.

Our study reveals the existence of a band of proneural activity. The PNCs are regions of elevated proneural activity in this band, rather than discrete clusters. In the band, the Notch pathway exerts an additional novel function, which defines the extent of the PNCs. In the absence of Notch function, most cells in the proneural band accumulate high levels of proneural activity that allows them to become SOPs. Thus, the pathway suppresses the proneural activity and the SOP fate in cells located between the PNCs in the proneural band. The short range of the Notch signal indicates that it is probably local mutual signalling among direct neighbours that generates the necessary Notch activity (mutual inhibition). The expression of Dl and Ser and the overall activity of Gbe+Su(H) (with exception of the halos) is unchanged in the absence of Ac and Sc. This suggests that the widespread activity of Notch in the notum that prevents most cells in the proneural band to become SOPs is not influenced by the proneural factors. It provides a baseline activity of Notch that suppresses the proneural activity in the band to prevent the formation of ectopic SOPs.

The presented results indicate that a subgroup within the PNCs exists, which is operationally defined via the requirement of the activity of Neur. The existence of a subgroup has previously been suggested on basis of experiments with a temperature sensitive allele of Notch [34]. These data and the ones presented here, suggest that the cells of the subgroup require Notch activity that is stronger than the baseline activity to be inhibited from adopting the SOP fate. This increase in activity is generated by the nascent SOP through a Neur enhanced Dl signal: We here found that if only one cell in the subgroup is *neur* positive, it can prevent all other *neur* mutant members to adopt the SOP fate. Thus, initiating the expression of Neur first, is a critical step for a cell to adopt the SOP fate, since it allows a cell to strongly inhibit its neighbours. The inhibitory signal prevents the accumulation of sufficient proneural activity to also activate Neur in the neighbours. This inhibition is probably reflected in the observed halo of Gbe+Su(H) expression around SOPs. The findings are in good agreement with a previous study that showed that the level of Neur in a cell is a critical factor for the formation of the SOP of the mc [18].

Loss of Notch activity results in expression of Neur and a dramatic increase in proneural activity in all cells of the PNC (e. g. see [19]). Moreover, the nascent SOP, which contains the highest proneural activity, is the only cell that initiates Neur expression during normal development and expression of *neur* is abolished in *ac sc* mutant discs [23]. These data indicate, that high proneural activity is required for the expression of Neur. Thus, the cell in the subgroup with the highest proneural activity is the cell that will express Neur first. The expression of Neur enables it to inhibit its neighbours from adopting the SOP fate by suppressing their proneural activity.

Our data therefore indicate that two activities of Notch are present during SOP formation. One generated through mutual signalling, which is not regulated by Ac and Sc and is sufficient to inhibit all cells in the proneural band outside the *neur* subgroup to become SOPs. This signalling requires the ubiquitously expressed Mib1 and antagonises the activity of Ac, Sc and Da. However, there is residual activity of Notch in *mib1* mutants sufficient to prevent most cells from adopting the SOP fate. This residual activity is generated either independently of E3 ligases or by another unknown E3-ligase. In any case this component contributes to the baseline activity of the Notch pathway in addition to Mib1. The second activity on top of the baseline activity in the *neur* subgroup is generated by a Neur mediated strong signal from the nascent SOP. This signal suppresses the proneural activity of the other members of the *neur* subgroup. It is dependent on proneural activity, which initiates the expression of Neur. Thus, lateral inhibition is probably operating after the emerging SOP reaches a threshold of proneural activity. It serves to prevent the formation of supernumerary SOPs in the *neur* group and assures that other cells can generate the necessary SOP in case the selected one is lost.

How is the *neur* subgroup defined? We found that the PNCs are small in their beginning, comprising the number of cells typical for the subgroup. These cells probably also constitute the small groups of SOPs observed in early third instar discs mutant for *Psn*. It is likely that E(spl)m8-SM expression defines this subgroup since we show here that it is expressed in a small group of cells from which the SOP arises. This construct contains only one E box, the binding sites for Ac and Sc, and response to high proneural activity [35]. We therefore believe that the cells of the early PNC are the *neur* group and possess the highest proneural activity.

During normal development, a cell with more proneural activity is already recognisable at the early phase of the PNCs. This suggests the existence of a pre-selection mechanism that assures that one cell in the *neur*-subgroup is advanced in its development. Evidence for such a mechanism has been also previously found during rescue experiments studying the function of the proneural genes Ac and Sc [36]. We have here obtained additional experimental evidence for this pre-selecting mechanism: In *neur* clones one of the cells is advanced in its development towards the SOP fate. Moreover, clonal analysis of *kuz* and *Psn* mutants revealed that wildtype cells at positions in the PNC where the SOP arises cannot be prevented from adopting the SOP fate, even if a mutant SOP that cannot be inhibited (e. g. *kuz* mutant), is its neighbour. We have shown that the mutant cells can generate a strong inhibitory Notch signal. This indicates that the pre-selecting mechanism renders the wildtype SOP immune to the signal. The nature of this mechanism is not clear, nor whether it is always the same cell in a cluster that is selected.

### The cells of the imaginal discs are constantly inhibited from adopting the SOP fate

Recent work demonstrated that in the eye disc a regulatory loop between Da and Emc assures correct expression of both factors and results in their complementary expression [5]. Consequently, loss of *emc* function results in an increase of expression of Da. The consequences of this up-regulation for the proneural state of the mutant cells have not been investigated in detail. The work focused on the eye imaginal disc and revealed that a few of the mutant cells in clones could adopt the neural fate. The neural cells do not express Runt, a marker expressed in the normal neural cells. Thus, the loss of *emc* does not result in the complete determination of the neural fate. The state of the vast majority of the cells in clones remained unknown. We observed up-regulation of proneural activity in *emc* clones already in early third instar wing imaginal discs, indicating that it is an immediate reaction to the loss of *emc* function. Some of these cells progress to become SOPs. The increase in proneural activity was also observed in *emc* clones of the leg disc. Thus, the cells of imaginal discs must be permanently inhibited from adopting a proneural state through the activity of Emc. It has to be pointed out that this situation is remarkably similar to that in the early vertebrate embryo, where all cells of the blastula adopt the proneural state unless they are inhibited through BMP signalling. The cells of the neural plate maintain the proneural state due to the presence of BMP antagonists (reviewed in [37]).

In the eye disc and during oogenesis expression of Emc is regulated by the Notch pathway [38], [39]. We failed to find evidence that supports a regulatory relationship between Emc and the pathway in the notum during SOP development, since the loss of *Psn* function did not affect the expression of EMC. However, it has been previously shown that the expression of Emc along the dorso-ventral boundary in the wing primordium depends on the activity of the Notch pathway [40]. This correlates well with our finding that this domain is independent of the activity of Da. However, the genetic network of the wing is significantly different from that in the notum. For example Notch signalling induces the expression of Wg along the D/V boundary. However, its expression in the proximal wing and in the notum is independent of the activity of the Notch pathway. This appears to be true also for the different domains of expression of Emc.

### The function of tissue specific proneural genes and Da

We here found that the function of *ac* and *sc* is dispensable for bristle development in the absence of *emc* function. How is the SOP fate initiated in these *emc ac sc* triple mutant cells? We believe that the activity of Da is sufficient for SOP development in this situation for the following reasons: 1. Da is expressed ubiquitously and is required for the formation of all external sense organs [41]. 2. Strong over-expression of Da induces bristle formation in cells that lack the whole AS-C [42], [43]. In contrast, over-expression of Sc fails to induce SOP formation in the absence of Da [43]. 3. Da can form homodimers that bind to the same DNA target sequences as Ac/Da and Sc/Da heterodimers in bend-shift assays [44]. 4. Loss of *emc* activity increases the activity of Da [5]. We here show that this increase is independent of the activity of Ac and Sc. 5. Our results show that Da regulates the expression of *sca* independently of Ac and Sc. 6. It has been shown that the mammalian homologue of Da, E2A, acts without its class II partners during B-cell development (reviewed in [45]). Thus, it is likely that in the absence of function of *emc, ac* and *sc*, Da forms active homo-dimers that initiate the required neural program.

While it is clear that the activity of Ac and Sc is required during normal development, the formation of normal bristles in their absence after concomitant loss of *emc* function raises the question about their function. Our data suggest that an important function is the neutralisation of Emc through formation of heterodimers with it or with Da. This releases Da from inactive heterodimers with Emc. The neutralisation of Emc by Ac and Sc, which are expressed in precise spatial and temporal regulated patterns, allows the differentiation of neural precursors at the correct position and time. The recent finding that a Sc variant without its transactivation domain is fully active fits well to this view of the function of Ac and Sc [43]. Thus, through their intricate and dynamic expression, Ac and Sc and other tissue specific proneural factors determine when and where a neural precursor cell develops. In this view the function of the tissue-specific proneural genes of *Drosophila*, is similar to that in mammals where their orthologs also promote differentiation of neural precursors in a proneural field, the neural plate, at correct positions and time.

### The determination of the SOP of the MC

**Figure 8:**
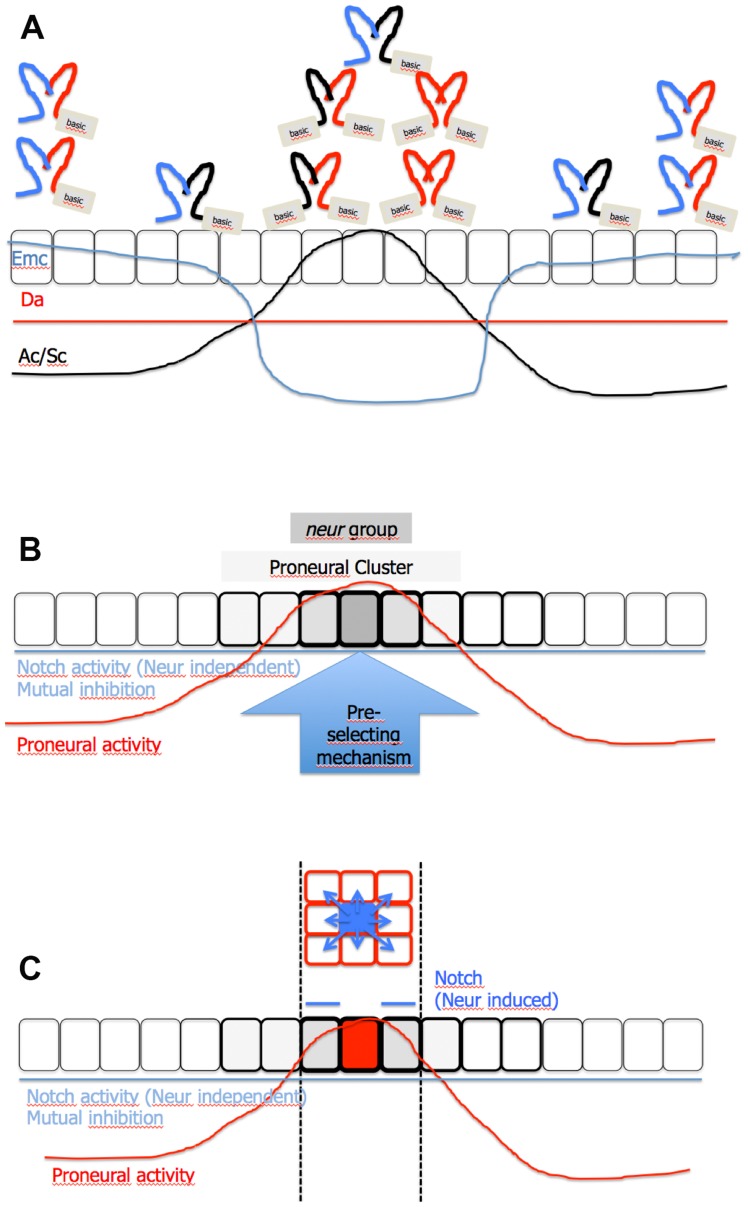
Model of SOP selection. (A) The proneural band in the notum is defined by differential expression of Emc. In valleys of Emc expression the PNCs are defined through the expression of Ac and Sc (black line). The proneural activity in the proneural band is a result of the baseline activity of the Notch pathway (produced by mutual inhibition), expression of Ac and Sc and expression of Emc. In regions of high Emc levels, Ac and Sc are absent and cells fail to reach sufficient levels of proneural activity, also due to the additional presence of the activity of the Notch pathway. As a result, Da forms inactive heterodimers with Emc. In regions where expression of Ac and Sc is initiated, expression of Emc is low. Moreover, Ac and Sc is expressed in these regions and form inactive dimers with Emc and therefore neutralise its negative effect on Da. The released Da can form active homo- and Da/Ac/Sc heterodimers to create high proneural activity. (B) The baseline activity of the Notch pathway suppresses the proneural activity also in the PNC and therefore restricts the ability to become a SOP to the *neur* subgroup. (C) In the subgroup the proneural activity is above a threshold level that enables cells to become SOPs. However, a pre-selecting mechanism favours the cell at the correct position to become the SOP, because it enables the cell to reach the threshold level of proneural activity to activate the expression of Neur first. The expression of Neur enables the nascent SOP to send a strong inhibitory signal through Dl to its neighbours. For further information, see S8 Fig.

Based on our results, we suggest a working model for the selection of the SOP of the MC (Fig. 8A–C): The differential expression of Emc defines a proneural band in the notum with changing proneural activity. The PNCs in this band are determined and positioned through the cluster-like expression of Ac and Sc, which increases the proneural activity at these positions. A baseline of activity of the Notch pathway generated by mutual inhibition prevents cells between the PNCs to accumulate high levels of proneural activity. In addition, it prevents cells located in the PNC, but outside the *neur* group, to accumulate high proneural activity required for adopting the SOP fate.

In the PNCs, expression of Ac and Sc neutralise Emc. Consequently, the proneural activity increases dramatically, since the released Da can form homodimers and/or heterodimers with Ac or Sc. The cells of the initial small PNCs later constitute the *neur* subgroup. The cells of this subgroup have the highest level of proneural activity and experience this activity also for the longest time. Within this subgroup a cell is pre-selected to become the SOP by a so far unidentified mechanism. Hence, it is the first to reach the threshold level of proneural activity required to initiate the expression of Neur. The expression of Neur enables it to efficiently inhibit the other cells of the subgroup through lateral inhibition. As a consequence these cells never accumulate sufficient proneural activity to activate Neur expression and to become a SOP. The strong signal also further activates the expression of Brd proteins that inhibit the activation of Neur, which might be accidentally activated weakly in one of the neighbours [46]. This activation contributes to the precision of determination process. Thus, a combination of mutual and lateral inhibition mediated by the Notch pathway operates in the PNC during the determination of the SOP. Only the lateral inhibition component depends on proneural activity through transcriptional activation of expression of Neur. For further information and how the model can explain the phenotypes of *neur* and *mib1* mutants, see (S8D-F Fig.) and the corresponding figure legend.

The model differs from the lateral inhibition model in the following points: No feedback loop between expression of Dl and proneural activity and, hence, no differential Dl expression is required. Instead the future SOP is pre-selected and advanced in its development. Subgroups within a proneural band defined through its requirement of Neur exist. In this subgroup the activation of the expression of Neur is critical for SOP development since it enables a cell to potently inhibit its neighbours. The pre-selection mechanism favours a cell at the right position to initiate the expression of Neur before the others of the Neur group and therefore secures its development as SOP. Moreover, the existence of mutual signalling explains the inhibition of cells in the proneural band outside the subgroup without the necessity of signalling of Dl over longer distances.

## Materials and Methods

### *Drosophila* genetics

UAS lines: UAS *Flp* (Bloomington stock collection). Gal4 lines: *ptc*Gal4 [47], *ci*Gal4 [48]. Other lines: Gbe+Su(H)-lacZ [20], NRE-pGr [21], Gbe+Su(H)-GFP [49], E(spl)m8-SM-GFP [22], YFP-Emc ([33]), *sca*-lacZ (Bloomimgton stock collection), *neur^A101^*-lacZ (Bloomington stock collection), DCE-GFP [29], UAS emc-RNAi (VDRC, line #100587), *tub*. rab7-YFP [50].

Mutants: *Psn^C1^ FRT2A* (null allele; [51]), *emc^AP6^* FRT80 [5] and *emc^1^* FRT80 (Bloomington stock collection #5532), *aph-1^D35^* FRT40A [52], *nic^A7^* [53], *sc^10.1^* (Bloomington stock collection), *mib1^2^* and *mib1^3^* [24], *mib1^EY09780^* [25], *neur^1^* FRT 82B (Bloomington stock collection, a gift from C. Delidakis), *Dl^rev10^ Ser^RX22^* FRT82B [54], *kuz^ES24^* FRT40A [55].

### Clonal analysis

Clones were generated with the FLP/FRT or MARCM system [56] and induced at the first larval instar (24-48h after egg laying) by applying a 1h heat shock (37°C). Alternatively, the clones were induced using a UAS *Flp* construct driven by *ptc*Gal4 in some of the cases of the *neur* and *emc* clones as indicated in the figures or figure legends. Flies were raised at 25C.

### Antibody staining and microscopy

Antibody staining was performed according to standard protocols. Primary antibodies used: mouse anti-Wg (4D4), mouse Dl antibody against the extracellular domain (C594.9B), anti Hnt (1G9), anti NICD (C17.9C6), anti NECD (C458.2H), 22C10/futsch antibody and anti Cut (2B10). All antibodies were purchased from the Developmental Studies Hybridoma Bank (DSHB). Anti Ser antibody (gift of Elisabeth Knust, [57]), anti-Da [41], Fluoro-chrome conjugated secondary antibodies were purchased from Invitrogen/Molecular Probes (Dianova, anti-gp). Images were obtained with a Zeiss AxioImager Z1 Microscope equipped with a Zeiss Apotome.

### Staging of discs

For staging of discs during pupal stages white pupae (0–1 h apf) were selected and staged accordingly. For larval discs the emerged Hnt positive SOPs were used in combination with the description of the emergence of SOPs by Huang et al. (1991).

## Supporting Information

S1 Fig The model of lateral inhibition during selection of the SOP. (A) Two cells of a PNC are shown. The right one will become the SOP. All cells of a PNC initially express similar levels of proneural activity and therefore mutually inhibit each other from adopting the SOP fate through Dl/Notch signalling. A small difference in activity of the proneural factors, which is generated by differential expression of Emc results in an initially small difference in proneural activity. Since the proneural activity is responsible for expression of Dl, the cell with higher proneural activity increases its expression of Dl and consequently inhibits its neighbours stronger and further down-regulates proneural activity and Dl expression in them. This loop of proneural activity, Dl expression and Notch activity amplifies small differences of proneural activity among cells of the PNC and transforms it into an all or nothing situation: The result is one cell with high proneural activity and high Dl expression that becomes the SOP (red arrow) and neighbours with eventually insufficient proneural activity that switch fate to become epidermoblasts. The SOP arises at positions of the lowest Emc expression and, hence, highest initial proneural activity. Note, that the lateral inhibition model predicts changes in the expression of Dl and the activity of the Notch pathway during the selection process. (a) If the nascent SOP contacts remotely located cells through filopodia, it also inhibits these cells from becoming a SOP through the described loop. (B) A cell in a PNC that has lost its ligands is unable to inhibit its neighbours (redly framed cell) and to take part in the loop. However, it can still receive the inhibitory signal. Therefore, it fails to accumulate sufficient proneural activity to become a SOP, even if it is located at the position where the SOP normally arises (case 1). (b) A mutant cell should also be prevented adopting the SOP fate by a SOP located more than one cell diameter, if the filopodia of this SOP deliver the inhibitory signal. Note that the mutant cell in (b) is the redly framed cell. (C) Conversely, a cell that has lost the Notch receptor cannot be inhibited by its neighbours and should accumulate sufficiently high levels of proneural activity to become a SOP and inhibit its neighbours, even if it is not located at the position where the SOP arises normally. Thus, it should also inhibit the formation of SOPs at the normal position if located there. The range of inhibition by this mutant SOP should also increase through the filopodia. (c) Consequently, also cells located further away should be inhibited to become SOPs, even if located at the position the SOP normally arises.
(TIFF)

S2 Fig (A–G) Comparison of expression of the ligands Dl and Ser relative to the Notch activity marker Gbe+Su(H)-lacZ. It reveals that the domains of Dl are shifted relative to the peaks of Gbe+Su(H) expression. The pattern of Ser overlaps more with that of Gbe+Su(H). The white arrow in (C–G) highlights the position of expression of stripe 3 of Gbe+Su(H). The comparison of (E–G) shows that Dl expression is low, while that of Ser is high in this region. The yellow arrow in (B, E) points to stripe-like domain 2 of Gbe+Su(H) expression. It highlights the fact that the domain is shifted relative to that of Dl expression. (H, I) Intracellular distribution of Dl and Rab7. (H) z-section of the region of the DC PNC. (I) Frontal view. The arrowhead points at the pDC position where the SOP is already determined indicated by the strong expression of *sca*-lacZ and the enlargement of the nucleus. The arrow points to an anteriorly located cell that has slightly increased levels of *sca* expression at the aDC position indicating that it initiates SOP development. The panels reveal that there is no difference in distribution and expression of Dl among the cells of the PNC during the selection of the SOP. They further reveal that the majority of Dl positive vesicles are positive for Rab7, indicating that they are maturing endosomes. (J) Expression of Dl and Hnt in *sc^10.1^* discs. Hnt and Dl are shown in the same channel in since both primary antibodies are derived from the same species (mouse). The proteins can be discriminated due to their different subcellular distribution (Hnt in the nucleus vs Dl in the cytosol and membrane) and the nuclear Hnt signal in SOPs is absent in *sc^10.1^* mutant discs. The expression of Dl is unchanged in *sc^10.1^* mutant discs, which lack the function of *ac* and *sc* (compare with E). However, Hnt positive SOPs are absent. White scale bar 50µm; cyan scale bar: 10µm.
(TIF)

S3 Fig The emergence of the SOPs of the DC cluster. (A–H) Discs of the third larval instar ordered according to increasing age from left to right. (A^1–4^, B^1–4^, C^1–4^, D^1–4^, E^1–4^, F^1–4^, G^1–4^, H^1–4^) A magnification of the area of the DC cluster of the discs shown in (A–H). The expression of *sca*-lacZ is shown to reveal the proneural activity in (A^4^, B^4^, C^4^, D^4^, E^4^, F^4^, G^4^, H^4^), that of the DC-E to highlight the DC cluster (A^3^, B^3^, C^3^, D^3^, E^3^, F^3^, G^3^, H^3^) and of Hnt to reveal the determined SOP (A^2^, B^2^, C^2^, D^2^, E^2^, F^2^, G^2^, H^2^). Two SOPs emerge from the DC cluster in a temporal sequence, with the pDC first followed by the aDC. The arrowheads in (C^4^, D^4^, H^4^) point to the cell with increased expression of *sca*-lacZ, which probably becomes the SOP. The arrowhead in (C^4^) points to the cell with increased *sca*-lacZ expression, which will become the pDC SOP. It has not initiated Hnt expression yet (see C^2^). The comparison with the expression of the DC-E reveals that it belongs to a group with higher expression of DC-E (C^3^, arrowhead). The arrowhead in (H^1–4^) highlights the aDC SOP that has just initiated Hnt expression (H^2^). As expected it expresses *sca*-lacZ stronger than its neighbours (H^4^) and arises among cells with higher DC-E expression (H^3^). White scale bar 50µm; cyan scale bar: 10µm.
(TIF)

S4 Fig Emergence of SOPs in PNCs of *Psn* mutant discs. The arrow and arrowheads in (A–E) highlight the DC and aPA/tr1 PNCs respectively, the yellow arrow points to the SC cluster. Maximum intensity projections are shown. The discs are all in the third larval instar stage and increase in age from (A) to (E). The comparison of the discs indicates that in the absence of Notch activity, one or a small group of cells adopt the SOP fate first. This indicates that these cells have higher proneural activity than the rest of the cells of the PNC. Note, that during further development, the PNCs increase and more cells adopt the SOP fate. These additional SOPs are added at the periphery to the already existing ones. Thus, the cells in the centre of the PNC are the cells with the highest competence to become SOPs. The cells contain the highest *sca* expression and therefore the highest proneural activity. (A, A′, A′′, A′′′) An early third instar discs bearing *sca*-lacZ expressing PNCs. At this time expression of Hnt is absent among cells of the PNC, indicating that they have not adopted the SOP fate yet.) A disc where none of the cells of the highlighted PNCs has adopted the SOP fate yet. (B–E′′′) The first SOPs are observed in the tr1/APA PNC (arrowhead). The next PNC where cells become SOPs is the DC PNC (arrow), followed by the SC one (yellow arrow). White scale bar 50µm; cyan scale bar: 10µm.
(TIF)

S5 Fig Notch signalling in *emc* mutant territories. (A–D) Expression of Gbe+Su(H) and Hnt in *emc* null mutant clones induced by *ptc*Gal4 UAS *Flp*. The clones are labelled by the absence of RFP. As can be seen in the large clone outlined in yellow in (C, D), no obvious change of expression of Gbe+Su(H) can be observed. However, a halo can be observed around the ectopic SOPs (arrows in A–D). (E–K) Expression of Dl in a disc bearing *emc*-clones. The clones are labelled by the absence of RFP. (E–G) The expression is unaffected by the loss of *emc*. The arrow points to a large clone that includes one of the stripe like domains of Dl expression. It is unaffected by the loss of *emc* function. (H–K) Closer examination of expression of Dl in hsFlp induced *emc* mutant cell clones. The arrow and arrowhead in (H) highlight some of the clones that cause ectopic expression of *sca*. These clones are shown at higher magnification in (I–K). Yellow scale bar: 250µm; white scale bar 50µm; cyan scale bar: 10µm
(TIF)

S6 Fig (A) Comparison of the expression of YFP-Emc and *emc*-lacZ. (A′-A′′′) Magnification of the region highlighted with the arrow in (A). The arrowhead in (A′-A′′′) points to a SOP. The comparison reveals that in contrast to YFP-Emc, *emc*-lacZ is strongly expressed in the SOP. Moreover, the valleys of expression of YFP-Emc are broader than in the case of *emc*-lacZ. (B-h) Comparison of expression of YFP-Emc with *sca*-lacZ during the third larval instar. Emc is expressed in all cells of the wing discs at different levels in different regions. The expression of Emc is dynamic and eventually comprises five domains of high expression with variable size. The comparison of the expression of Emc and *sca* reveals that: 1. The PNCs are positioned in regions of low Emc expression and surrounded by domains of high expressions. 2. No obvious differential expression can be recognised among cells of PNCs that could predict where the SOP will form. In the following the expression of different stages of the third larval instar are described in detail to illustrate the conclusions. In general, Emc is expressed in all cells of the imaginal discs at varying levels. In the following only the domains of high expression are mentioned. (B-B′′′) Expression of Emc precedes that of *sca* in the notum of early third larval instar discs. At this time two domains of high Emc expression can be recognised. The arrowhead in (B) points to a single *sca*-expressing cell that emerges at the position of the dR SOP of the hinge region. This indicates that one cell in this emerging cluster is advanced in its development towards the SOP fate. No Hnt expressing cells can be detected, indicating that no cells have adopted the SOP fate. (C-C′′′) A slightly older disc than shown in (B). Emc expression still comprises the two domains of high expression, while *sca* expression is initiated weakly throughout most of the notum (asterisk). Note, that now several *sca* cells expressing can be observed at the dR PNC (arrowhead in C). Hnt expression and therefore SOPs are absent (C′). (D-D′′′) Shortly after initiation, a single strongly *sca*-lacZ expressing cell, which is probably the future SOP, is recognisable (D, arrow). The arrowhead highlights the region of the dR PNC. No Hnt is detectable at both positions (D′′). A third domain of strong Emc expression has appeared (D′). (E-E′′) Magnification of the notal region of a slightly older disc than shown in (D-D′′′). A valley of Emc expression between the three domains of strong expression can be recognised that resembles the shape of the proneural band. In this valley the PNCs emerge. The DC and tr1/aPA PNCs are recognisable (arrowhead and arrow, respectively). They are small at this time, but the tr1/aPA PNC already contains a cell with elevated *sca* expression (arrow in (E′′)). (e, e′) z-sections of the disc shown in (E-E′′) (e) A z-section along the A/P axis in the regions of the DC cluster indicating that it is located adjacent to the large domain 1 of high Emc expression. It reveals that several *sca* expressing PNC cells express similar levels of Emc. (e′) A z section along the P/D axis at the level of the DC PNC. Again the cells of the PNC express similar levels of Emc. Altogether, no clear difference of Emc expression indicative of SOP determination can be recognised in both z-sections. (F-F′′′) The initial PNCs enlarge in slightly older discs and single cells with higher expression of *sca* become recognisable, which will become SOPs (arrowhead and arrow in F′′′). Note, that the determination has not been complete, since the nascent SOPs do not express Hnt yet (F′′). Emc expression comprises three domains of high expression (F′). (g, g′) z-section along the A/P axis at the level of the Dc and tr1/aPA PNCs highlighted by the arrow and arrowhead respectively. They reveal that the nascent SOP (highlighted by the arrowhead and arrow) expresses levels of Emc indistinguishable from its neighbours. The numbers highlight the domains of high Emc expression. (H-H′′′) A late third instar disc with developed PNCs and Emc expression. The PNCs are maximally enlarged and Emc is expressed in five domains of high expression with different size (H, H′). The SOPs are determined and express Hnt (H′′). The PNCs are located in the regions of low expression (H′′′). (h-h′′′) Magnification of the region of the pDC cluster of the disc shown in (H-H′′′). It reveals that the expression of Emc is switched off in the determined pDC SOP (arrow). Note that this cannot be recognised in the *emc*-lacZ line. White scale bar 50µm; cyan scale bar: 10µm.
(TIF)

S7 Fig (A-A′′′) Expression of YFP-Emc is unchanged in *sc^10.1^* wing discs (compare with suppl. Fig. 6H-H′′). The arrow in (A′′′) points to the Hnt positive tracheal cells which are not relevant for SOP development. (B-B′′) Expression of *sca*-lacZ and Hnt in a *sc^10.1^ Psn^C1^* mutant wing disc. The arrows highlight the elevated expression of *sca*-lacZ and Hnt in the remaining PNCs. The arrowhead in (B′-B′′) highlights the Hnt positive tracheal cells that are not part of the disc proper. (C-C′′) Depletion of Da by expression of *da*-RNAi with *ptc*Gal4 results in the strong reduction of Emc expression (arrow). The arrowhead in (C′) and (C′′) points to the expression of Emc along the D/V compartment boundary, which is independent of Da. (D-D′) Expression of YFP-Emc in a *Psn* mutant disc. The comparisons with the expression of *sca*-lacZ reveals that the regions in the proneural band where the cells are most resistant to become SOPs are the regions where the cells express high levels of Emc (domains 4 and 5 of high expression, red and yellow arrows). White scale bar 50µm; cyan scale bar: 10µm.
(TIF)

S8 Fig Model of SOP selection. (A) The proneural band in the notum is defined by differential expression of Emc. In valleys of Emc expression the PNCs are generated by the expression of Ac and Sc. The proneural activity in the proneural band is a result of activity of the interplay of the Notch pathway, expression of Ac and Sc and expression of Emc. In regions of high Emc levels, Ac and Sc are absent. As a result, Da forms inactive heterodimers with Emc. In regions where expression of Ac and Sc is initiated, expression of Emc is low. Moreover, Ac and Sc form inactive dimers with Emc and therefore neutralise its negative effect on Da. As a result free Da can form active homo- and Da/Ac/Sc heterodimers to create high proneural activity. (B) The activity of the Notch pathway produced by mutual inhibition generate a baseline activity of Notch that restricts the ability to become a SOP to the *neur* group of the PNC. (C) In the *neur* group the proneural activity is above a threshold level that enables cells to become SOPs. However, a pre-selecting mechanism favours the cell at the correct position to become the SOP, because it enables the cell to reach the threshold level of proneural activity to activate the expression of Neur first. The expression of Neur enables the nascent SOP to send a strong inhibitory signal through Dl to its neighbours. (D) Possible scenario in *mib1* mutants. The activity of Notch generated by mutual inhibition is strongly reduced and consequently the proneural activity in the cells of the proneural band increases. As in the wildtype, the pre-selected cell of *neur* subgroup reaches the necessary proneural activity to initiate Neur expression first and inhibit its immediate neighbours. This results in an initial normal pattern of SOPs in the notum, although their formation is accelerated. With time, PNC cells further away from the pre-selected SOP eventually accumulate sufficient levels to express Neur over time and start to express Neur. This enables them to send a strong lateral inhibitory signal to its neighbours. In this way a pattern of SOPs is generated where all forming SOPs are separated by epidermoblasts. Hence, only few SOPs are generated in the PNC. Thus, due to the presence of the Neur mediated lateral inhibition, only a few ectopic SOPs can develop in the absence of Mib1. If a PNC comprises 30 cells and a SOP is surrounded by 8-9 cells, one would expect the emergence of 3-4 SOPs upon loss of *mib1* function. This is in good agreement with the observed phenotype of pharate adult *mib1* mutants [24]. (E) The loss of Neur results in the loss of lateral inhibition operating in the *neur* subgroup. Thus, the pre-selected SOP is unable to generate the potent inhibitory signal to inhibit its neighbours. Consequently, all cells in the *neur* subgroup eventually become SOPs. The cells outside the subgroup are still inhibited through the remaining baseline Notch activity generated through mutual signalling. (F) Complete loss of both E3 ligases results in loss of Notch activity and therefore causes all cells in the proneural band to eventually adopt the SOP fate over time. Note the model predicts that the cells the adopt the SOP fate first are the cells of the Neur subgroup and that SOP are sequentially added to this group over time from cells that start with lesser proneural activity. This is what we have observed in *Psn* mutant wing discs.
(TIF)
