# Supplementary figures and images for: A Re-examination of the Selection of the Sensory Organ Precursor of the Bristle Sensilla of Drosophila melanogaster
Source: PLoS Genet. 2015 Jan 8;11(1):e1004911. doi: 10.1371/journal.pgen.1004911 (PMC4287480; doi:10.1371/journal.pgen.1004911)

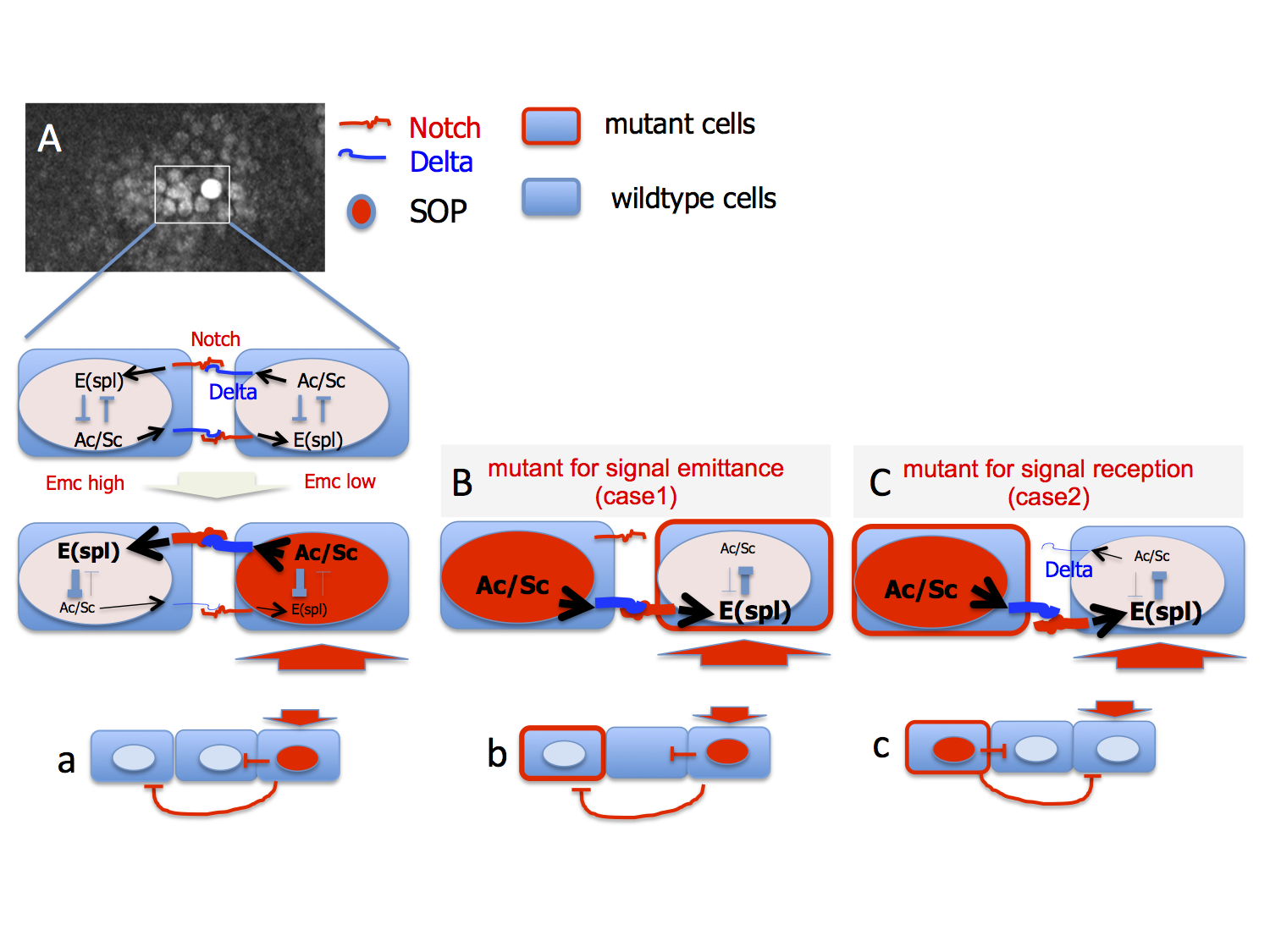

Supplement: S1 Fig — The model of lateral inhibition during selection of the SOP. (A) Two cells of a PNC are shown. The right one will become the SOP. All cells of a PNC initially express similar levels of proneural activity and therefore mutually inhibit each other from adopting the SOP fate through Dl/Notch signalling. A small difference in activity of the proneural factors, which is generated by differential expression of Emc results in an initially small difference in proneural activity. Since the proneural activity is responsible for expression of Dl, the cell with higher proneural activity increases its expression of Dl and consequently inhibits its neighbours stronger and further down-regulates proneural activity and Dl expression in them. This loop of proneural activity, Dl expression and Notch activity amplifies small differences of proneural activity among cells of the PNC and transforms it into an all or nothing situation: The result is one cell with high proneural activity and high Dl expression that becomes the SOP (red arrow) and neighbours with eventually insufficient proneural activity that switch fate to become epidermoblasts. The SOP arises at positions of the lowest Emc expression and, hence, highest initial proneural activity. Note, that the lateral inhibition model predicts changes in the expression of Dl and the activity of the Notch pathway during the selection process. (a) If the nascent SOP contacts remotely located cells through filopodia, it also inhibits these cells from becoming a SOP through the described loop. (B) A cell in a PNC that has lost its ligands is unable to inhibit its neighbours (redly framed cell) and to take part in the loop. However, it can still receive the inhibitory signal. Therefore, it fails to accumulate sufficient proneural activity to become a SOP, even if it is located at the position where the SOP normally arises (case 1). (b) A mutant cell should also be prevented adopting the SOP fate by a SOP located more than one cell diameter, [file pgen.1004911.s001.tif]

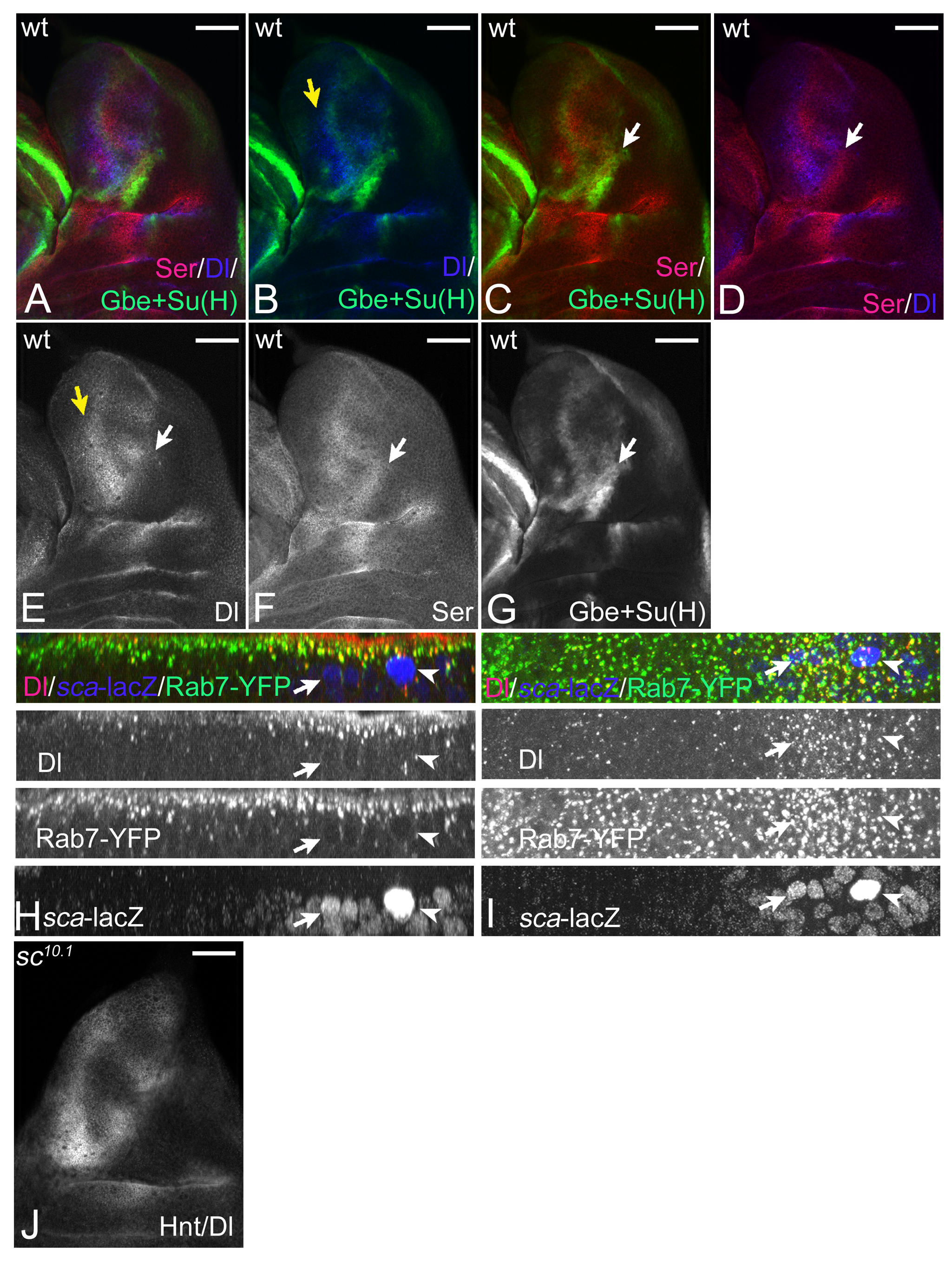

Supplement: S2 Fig — (A–G) Comparison of expression of the ligands Dl and Ser relative to the Notch activity marker Gbe+Su(H)-lacZ. It reveals that the domains of Dl are shifted relative to the peaks of Gbe+Su(H) expression. The pattern of Ser overlaps more with that of Gbe+Su(H). The white arrow in (C–G) highlights the position of expression of stripe 3 of Gbe+Su(H). The comparison of (E–G) shows that Dl expression is low, while that of Ser is high in this region. The yellow arrow in (B, E) points to stripe-like domain 2 of Gbe+Su(H) expression. It highlights the fact that the domain is shifted relative to that of Dl expression. (H, I) Intracellular distribution of Dl and Rab7. (H) z-section of the region of the DC PNC. (I) Frontal view. The arrowhead points at the pDC position where the SOP is already determined indicated by the strong expression of sca-lacZ and the enlargement of the nucleus. The arrow points to an anteriorly located cell that has slightly increased levels of sca expression at the aDC position indicating that it initiates SOP development. The panels reveal that there is no difference in distribution and expression of Dl among the cells of the PNC during the selection of the SOP. They further reveal that the majority of Dl positive vesicles are positive for Rab7, indicating that they are maturing endosomes. (J) Expression of Dl and Hnt in sc10.1 discs. Hnt and Dl are shown in the same channel in since both primary antibodies are derived from the same species (mouse). The proteins can be discriminated due to their different subcellular distribution (Hnt in the nucleus vs Dl in the cytosol and membrane) and the nuclear Hnt signal in SOPs is absent in sc10.1 mutant discs. The expression of Dl is unchanged in sc10.1 mutant discs, which lack the function of ac and sc (compare with E). However, Hnt positive SOPs are absent. White scale bar 50µm; cyan scale bar: 10µm. (TIF) [file pgen.1004911.s002.tif]

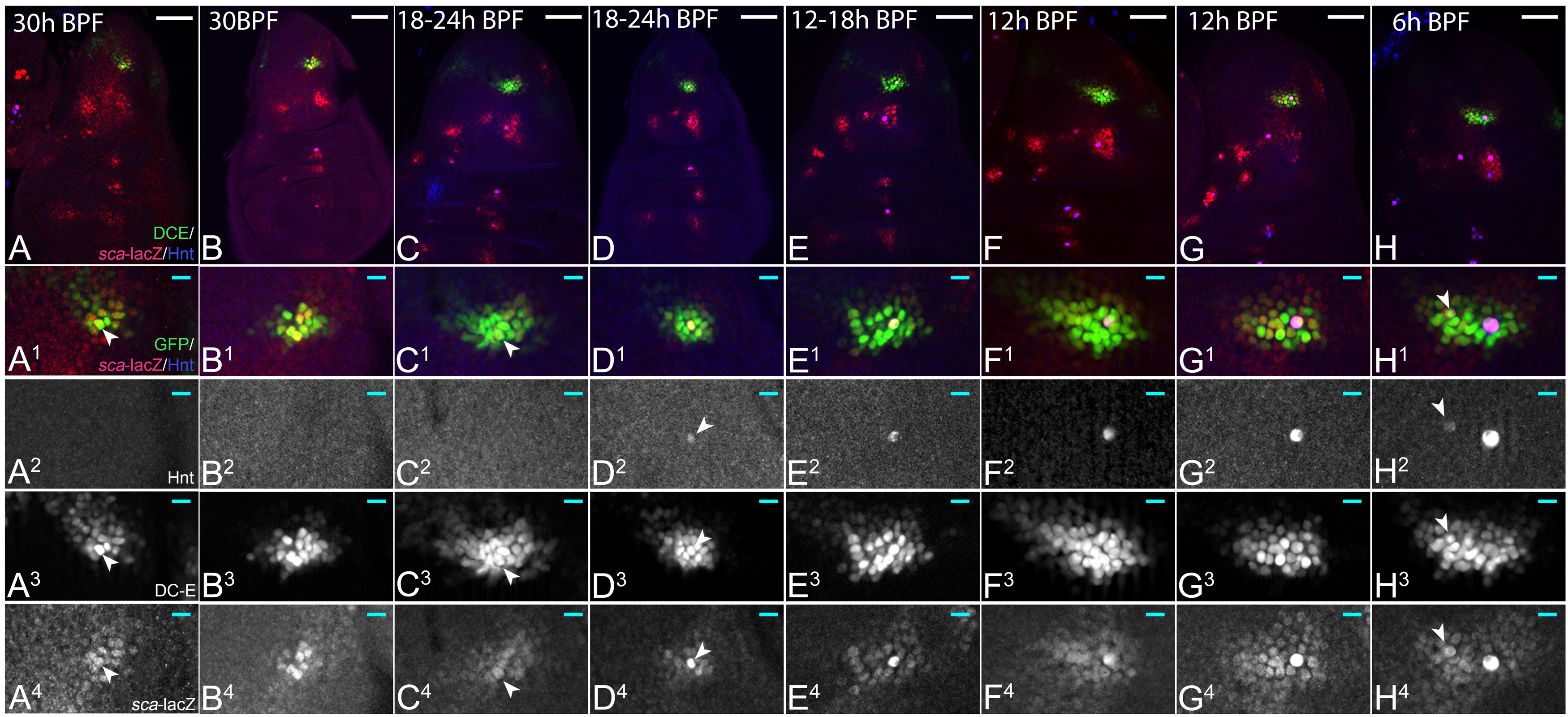

Supplement: S3 Fig — The emergence of the SOPs of the DC cluster. (A–H) Discs of the third larval instar ordered according to increasing age from left to right. (A1–4, B1–4, C1–4, D1–4, E1–4, F1–4, G1–4, H1–4) A magnification of the area of the DC cluster of the discs shown in (A–H). The expression of sca-lacZ is shown to reveal the proneural activity in (A4, B4, C4, D4, E4, F4, G4, H4), that of the DC-E to highlight the DC cluster (A3, B3, C3, D3, E3, F3, G3, H3) and of Hnt to reveal the determined SOP (A2, B2, C2, D2, E2, F2, G2, H2). Two SOPs emerge from the DC cluster in a temporal sequence, with the pDC first followed by the aDC. The arrowheads in (C4, D4, H4) point to the cell with increased expression of sca-lacZ, which probably becomes the SOP. The arrowhead in (C4) points to the cell with increased sca-lacZ expression, which will become the pDC SOP. It has not initiated Hnt expression yet (see C2). The comparison with the expression of the DC-E reveals that it belongs to a group with higher expression of DC-E (C3, arrowhead). The arrowhead in (H1–4) highlights the aDC SOP that has just initiated Hnt expression (H2). As expected it expresses sca-lacZ stronger than its neighbours (H4) and arises among cells with higher DC-E expression (H3). White scale bar 50µm; cyan scale bar: 10µm. (TIF) [file pgen.1004911.s003.tif]

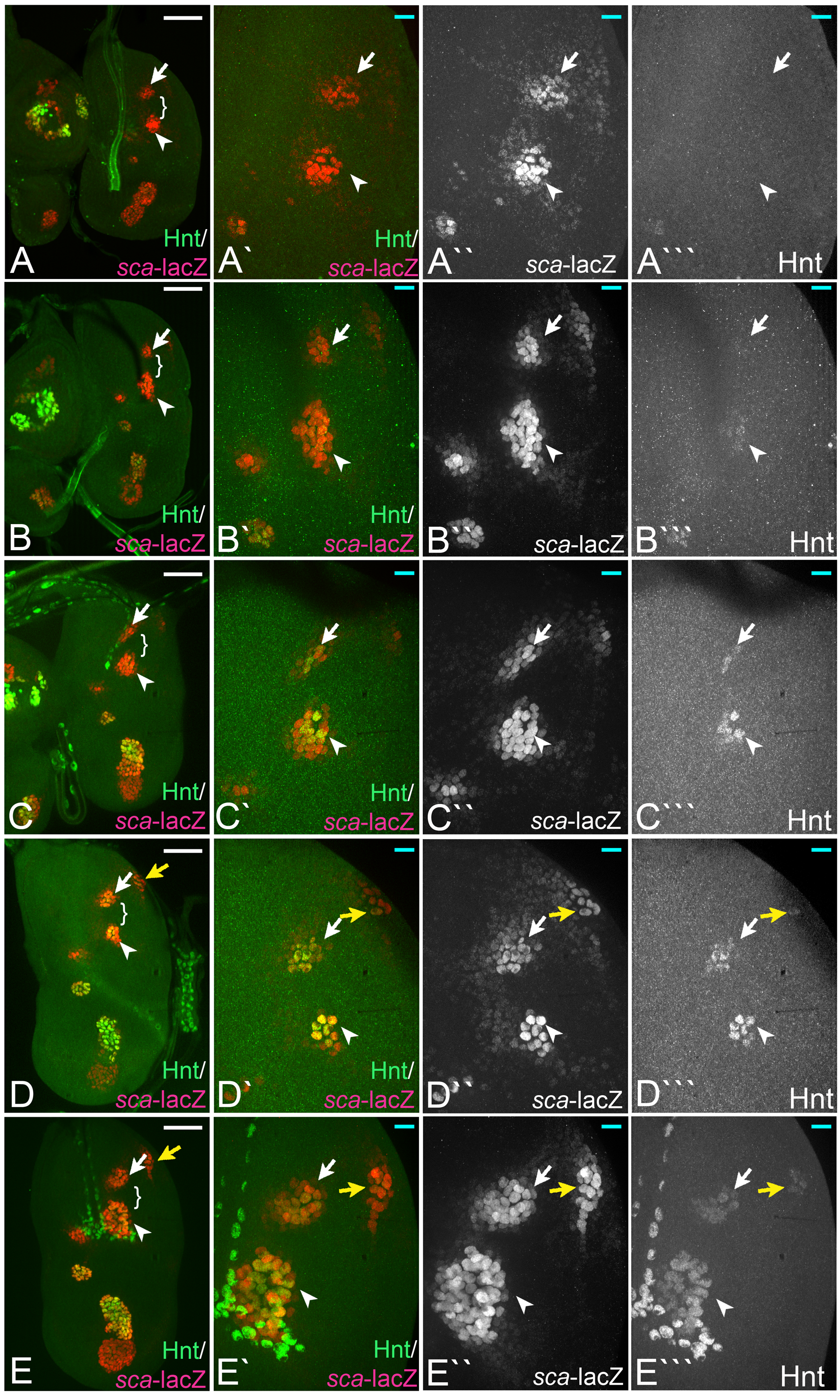

Supplement: S4 Fig — Emergence of SOPs in PNCs of Psn mutant discs. The arrow and arrowheads in (A–E) highlight the DC and aPA/tr1 PNCs respectively, the yellow arrow points to the SC cluster. Maximum intensity projections are shown. The discs are all in the third larval instar stage and increase in age from (A) to (E). The comparison of the discs indicates that in the absence of Notch activity, one or a small group of cells adopt the SOP fate first. This indicates that these cells have higher proneural activity than the rest of the cells of the PNC. Note, that during further development, the PNCs increase and more cells adopt the SOP fate. These additional SOPs are added at the periphery to the already existing ones. Thus, the cells in the centre of the PNC are the cells with the highest competence to become SOPs. The cells contain the highest sca expression and therefore the highest proneural activity. (A, A′, A′′, A′′′) An early third instar discs bearing sca-lacZ expressing PNCs. At this time expression of Hnt is absent among cells of the PNC, indicating that they have not adopted the SOP fate yet.) A disc where none of the cells of the highlighted PNCs has adopted the SOP fate yet. (B–E′′′) The first SOPs are observed in the tr1/APA PNC (arrowhead). The next PNC where cells become SOPs is the DC PNC (arrow), followed by the SC one (yellow arrow). White scale bar 50µm; cyan scale bar: 10µm. (TIF) [file pgen.1004911.s004.tif]

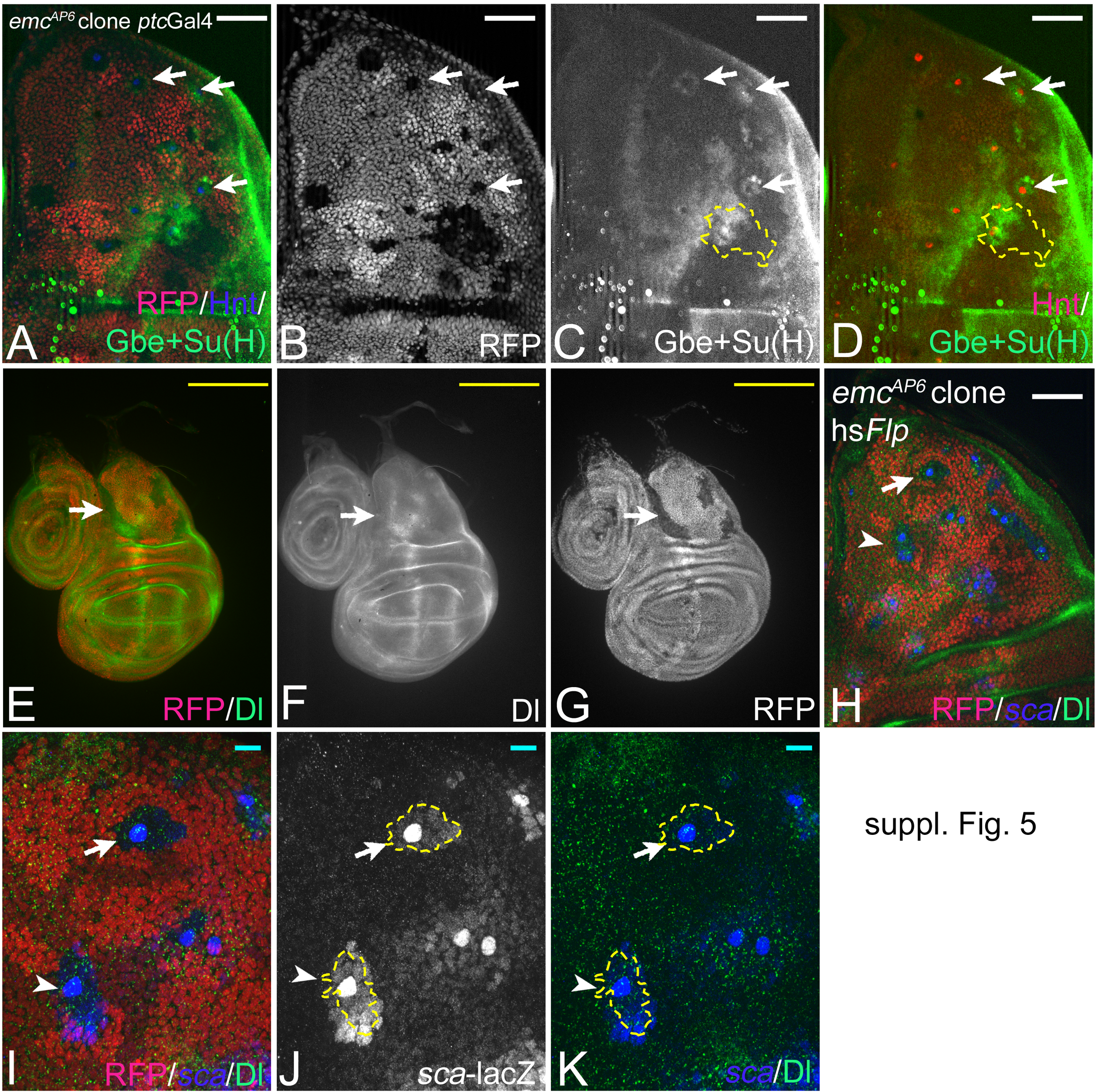

Supplement: S5 Fig — Notch signalling in emc mutant territories. (A–D) Expression of Gbe+Su(H) and Hnt in emc null mutant clones induced by ptcGal4 UAS Flp. The clones are labelled by the absence of RFP. As can be seen in the large clone outlined in yellow in (C, D), no obvious change of expression of Gbe+Su(H) can be observed. However, a halo can be observed around the ectopic SOPs (arrows in A–D). (E–K) Expression of Dl in a disc bearing emc-clones. The clones are labelled by the absence of RFP. (E–G) The expression is unaffected by the loss of emc. The arrow points to a large clone that includes one of the stripe like domains of Dl expression. It is unaffected by the loss of emc function. (H–K) Closer examination of expression of Dl in hsFlp induced emc mutant cell clones. The arrow and arrowhead in (H) highlight some of the clones that cause ectopic expression of sca. These clones are shown at higher magnification in (I–K). Yellow scale bar: 250µm; white scale bar 50µm; cyan scale bar: 10µm (TIF) [file pgen.1004911.s005.tif]

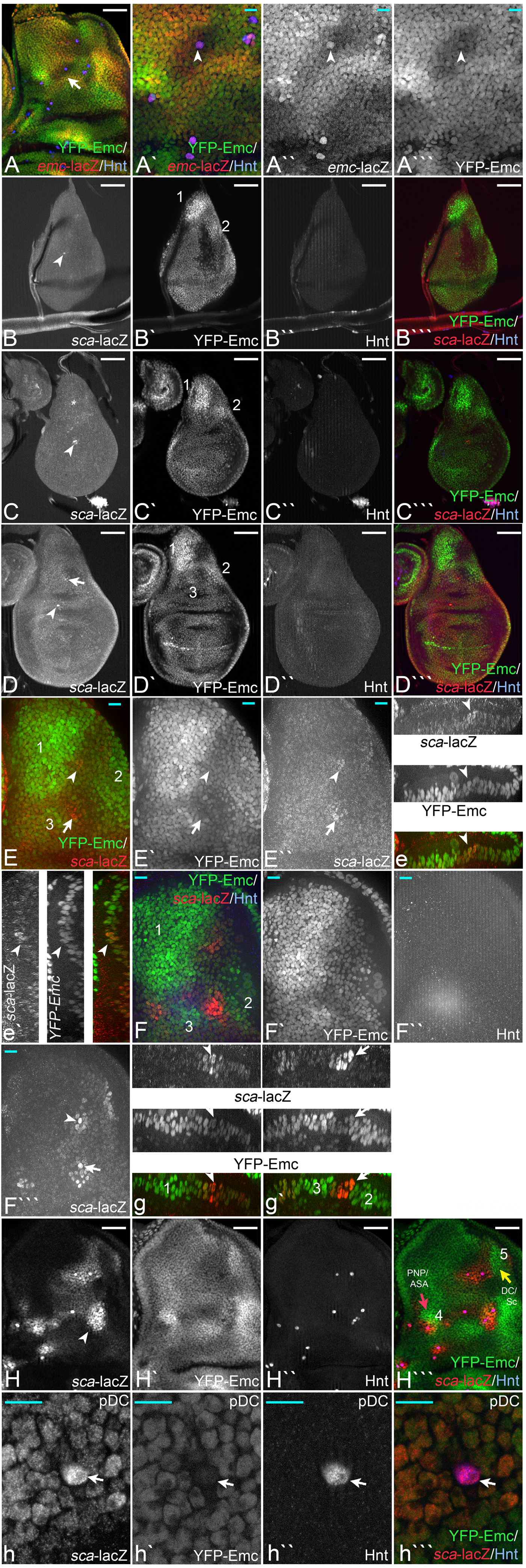

Supplement: S6 Fig — (A) Comparison of the expression of YFP-Emc and emc-lacZ. (A′-A′′′) Magnification of the region highlighted with the arrow in (A). The arrowhead in (A′-A′′′) points to a SOP. The comparison reveals that in contrast to YFP-Emc, emc-lacZ is strongly expressed in the SOP. Moreover, the valleys of expression of YFP-Emc are broader than in the case of emc-lacZ. (B-h) Comparison of expression of YFP-Emc with sca-lacZ during the third larval instar. Emc is expressed in all cells of the wing discs at different levels in different regions. The expression of Emc is dynamic and eventually comprises five domains of high expression with variable size. The comparison of the expression of Emc and sca reveals that: 1. The PNCs are positioned in regions of low Emc expression and surrounded by domains of high expressions. 2. No obvious differential expression can be recognised among cells of PNCs that could predict where the SOP will form. In the following the expression of different stages of the third larval instar are described in detail to illustrate the conclusions. In general, Emc is expressed in all cells of the imaginal discs at varying levels. In the following only the domains of high expression are mentioned. (B-B′′′) Expression of Emc precedes that of sca in the notum of early third larval instar discs. At this time two domains of high Emc expression can be recognised. The arrowhead in (B) points to a single sca-expressing cell that emerges at the position of the dR SOP of the hinge region. This indicates that one cell in this emerging cluster is advanced in its development towards the SOP fate. No Hnt expressing cells can be detected, indicating that no cells have adopted the SOP fate. (C-C′′′) A slightly older disc than shown in (B). Emc expression still comprises the two domains of high expression, while sca expression is initiated weakly throughout most of the notum (asterisk). Note, that now several sca cells expressing can be observed at the dR PNC (arrowhead in C). [file pgen.1004911.s006.tif]

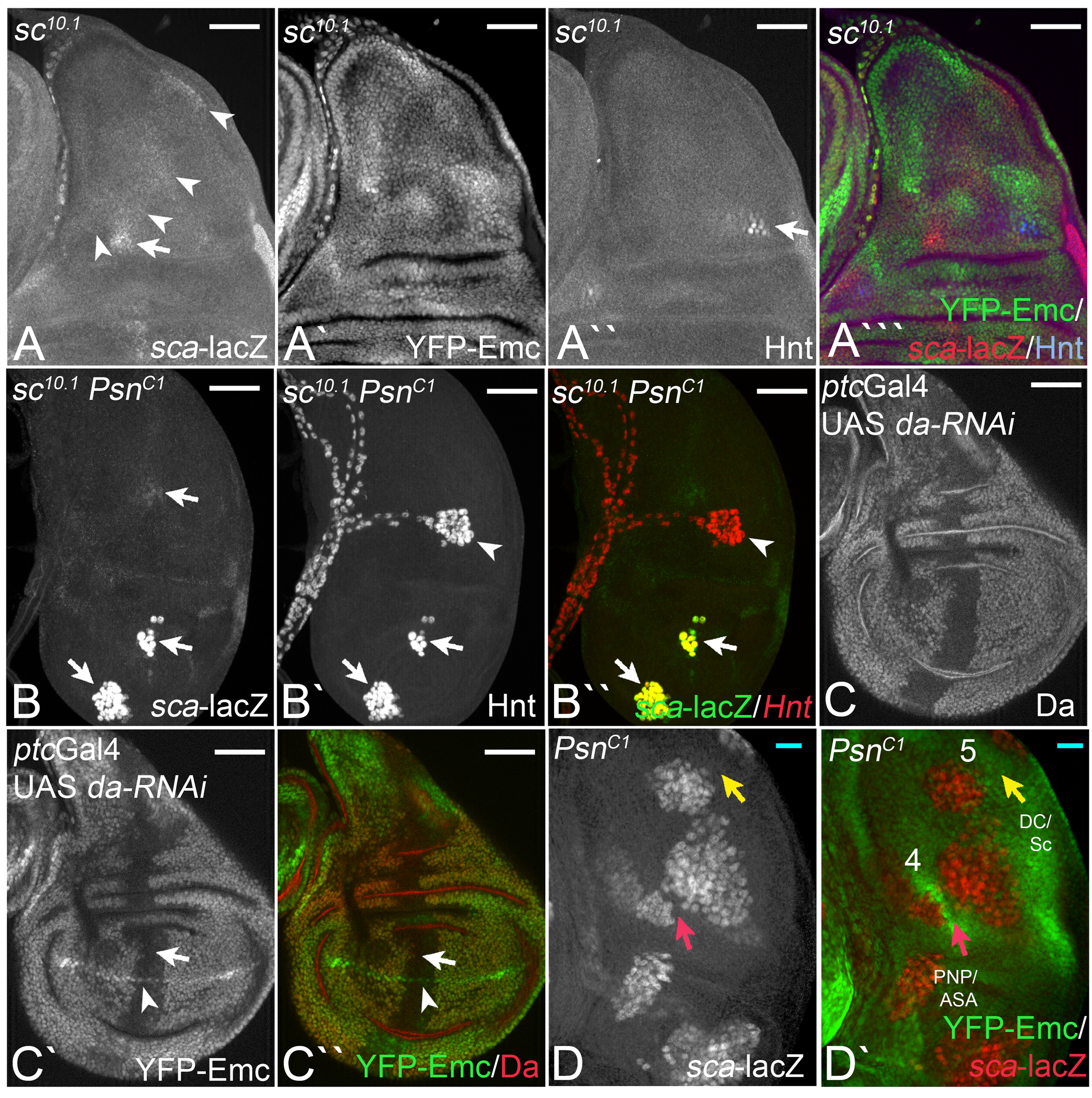

Supplement: S7 Fig — (A-A′′′) Expression of YFP-Emc is unchanged in sc10.1 wing discs (compare with suppl. Fig. 6H-H′′). The arrow in (A′′′) points to the Hnt positive tracheal cells which are not relevant for SOP development. (B-B′′) Expression of sca-lacZ and Hnt in a sc10.1 PsnC1 mutant wing disc. The arrows highlight the elevated expression of sca-lacZ and Hnt in the remaining PNCs. The arrowhead in (B′-B′′) highlights the Hnt positive tracheal cells that are not part of the disc proper. (C-C′′) Depletion of Da by expression of da-RNAi with ptcGal4 results in the strong reduction of Emc expression (arrow). The arrowhead in (C′) and (C′′) points to the expression of Emc along the D/V compartment boundary, which is independent of Da. (D-D′) Expression of YFP-Emc in a Psn mutant disc. The comparisons with the expression of sca-lacZ reveals that the regions in the proneural band where the cells are most resistant to become SOPs are the regions where the cells express high levels of Emc (domains 4 and 5 of high expression, red and yellow arrows). White scale bar 50µm; cyan scale bar: 10µm. (TIF) [file pgen.1004911.s007.tif]

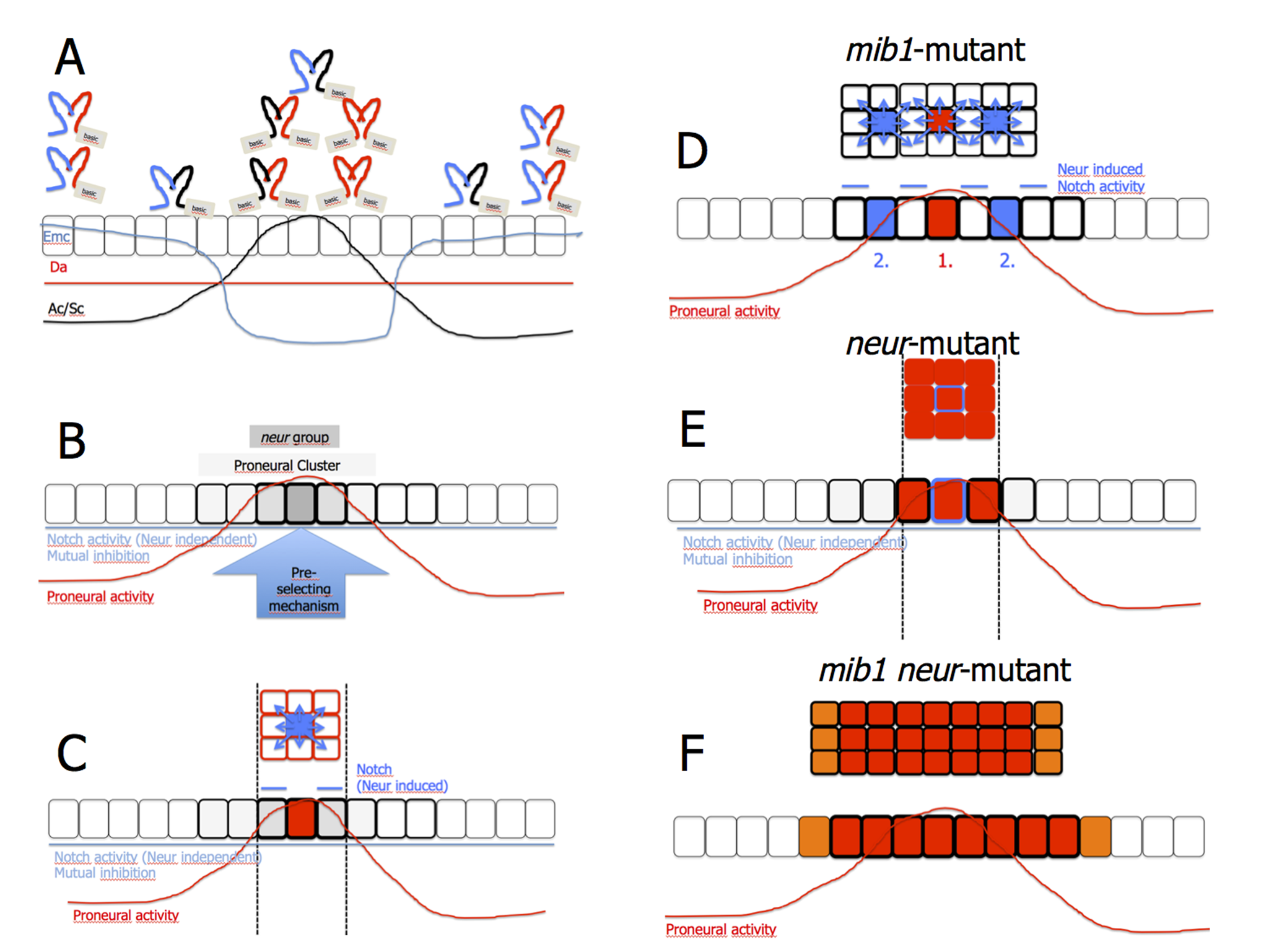

Supplement: S8 Fig — Model of SOP selection. (A) The proneural band in the notum is defined by differential expression of Emc. In valleys of Emc expression the PNCs are generated by the expression of Ac and Sc. The proneural activity in the proneural band is a result of activity of the interplay of the Notch pathway, expression of Ac and Sc and expression of Emc. In regions of high Emc levels, Ac and Sc are absent. As a result, Da forms inactive heterodimers with Emc. In regions where expression of Ac and Sc is initiated, expression of Emc is low. Moreover, Ac and Sc form inactive dimers with Emc and therefore neutralise its negative effect on Da. As a result free Da can form active homo- and Da/Ac/Sc heterodimers to create high proneural activity. (B) The activity of the Notch pathway produced by mutual inhibition generate a baseline activity of Notch that restricts the ability to become a SOP to the neur group of the PNC. (C) In the neur group the proneural activity is above a threshold level that enables cells to become SOPs. However, a pre-selecting mechanism favours the cell at the correct position to become the SOP, because it enables the cell to reach the threshold level of proneural activity to activate the expression of Neur first. The expression of Neur enables the nascent SOP to send a strong inhibitory signal through Dl to its neighbours. (D) Possible scenario in mib1 mutants. The activity of Notch generated by mutual inhibition is strongly reduced and consequently the proneural activity in the cells of the proneural band increases. As in the wildtype, the pre-selected cell of neur subgroup reaches the necessary proneural activity to initiate Neur expression first and inhibit its immediate neighbours. This results in an initial normal pattern of SOPs in the notum, although their formation is accelerated. With time, PNC cells further away from the pre-selected SOP eventually accumulate sufficient levels to express Neur over time and start to express Neur. This enables them to [file pgen.1004911.s008.tif]
